# Monthly and annual temperature extremes and their changes on the Tibetan Plateau and its surroundings during 1963–2015

**DOI:** 10.1038/s41598-018-30320-0

**Published:** 2018-08-08

**Authors:** Jin Ding, Lan Cuo, Yongxin Zhang, Fuxin Zhu

**Affiliations:** 1 Key Laboratory of Tibetan Environment Changes and Land Surface Processes, Institute of Tibetan Plateau Research, Chinese Academy of Sciences, Beijing, China; 2 University of Chinese Academy of Sciences, Beijing, China; 3 Center for Excellence in Tibetan Plateau Earth Sciences, Beijing, China; 4 Research Applications Laboratory and Climate and Global Dynamics Laboratory, National Center for Atmospheric Research, Boulder, Colorado, USA

## Abstract

In this study, the spatiotemporal distributions of monthly and annual temperature minima (T_min_) and maxima (T_max_), extreme T_min_ and T_max_, the highest (lowest) T_min_ (T_max_), frost day (FD), icing day (ID), summer day (SD) and tropical night (TR) at 112 stations and over the ten large river basins on the Tibetan Plateau and its surroundings (TPS) during 1963–2015 are examined. Mann-Kendall test is applied for the trends. The analyses show: the northwest experiences the hottest summer while the central TPS has the coldest winter and most frequent frost and icing days. The northwest (southeast) features the highest (lowest) monthly extreme temperature ranges. The northwest has the most frequent hot summer days, whereas the southeast has the least frequent frost and icing days. The entire TPS displays few tropical nights. Most stations show positive trends for all monthly and annual T_min_ and T_max_ variables. February displays the most positive trends for both monthly T_min_ and T_max_ variables while April shows the highest number of stations with decreasing trends in monthly T_max_. The trends of FD and ID are negative, whereas the trends of SU and TR are positive. Over river basins, the trends of monthly T_min_ are all positive and statistically significant and the trends of monthly T_max_ are all positive except for one negative trend and around 1/3 of the positive trends are statistically significant. Relatively larger increases in monthly T_min_ and T_max_ are noted for the cold season than the warm. The monthly and annual T_min_ variables increase more than T_max_ variables.

## Introduction

Extreme weather events such as heat waves, floods, droughts or storms have been shown to exhibit an increased frequency in recent decades^1–6^. Global climate models simulate a link between a warmer climate and changes in extreme weather events^7–9^, and there have also been observational evidences of this link^4,10^. Extreme weather events can lead to severe societal and economical impacts and the cost of extreme events has increased dramatically over the recent decades^11^. For instance, in 1980–2011, the total loss from major weather and climate disasters in U.S. was estimated at $881 billion, among which drought/heat waves contributed to a quarter of the total loss^12^. For China, the number of heat waves and extreme storm events has also increased during 1960–2013 while the number of extreme cold events has reduced due to the warming trend^13^. The economic losses caused by these and other extreme weather and climate events have been rising steadily during the past decades in China^13^.

Because of their significantly detrimental effects on society and natural ecosystems, extreme weather and climate events have been studied extensively in recent decades, of which temperature extremes are one of the most commonly examined due to (a) temperature records tend to be long and (b) projections of temperature tend to be more robust^14^. Changes in temperature extremes have been documented across the globe^2,3,15^ and over various regions specifically, for example, the Caribbean region^16^; Europe^17^; central to south Asia^18^; Central and South America^19^; the Middle East^20^; Canada^21^; Africa^22^; China^23^; Western U.S.^24^, Georgia^25^, Iran^26^, the Iberian Peninsula^27^, and Serbia^28^, though with large spatial variations regionally^29^ and/or asymmetric changes in temperature extremes^30,31^. It is expected that the spatiotemporal variations in temperature extremes could be significant over complex terrains such as the Tibetan Plateau (TP); however, while many studies focused on annual scale temperature extremes and using a handful of stations on the TP above certain elevations, systematic investigation based on monthly time series and including the surroundings of the TP has not been carried out as of this writing.

Temperature extremes and their changes have been shown to influence daily mortality in Spain^32^, tendencies of accidents in building facilities and workers’ accidents in Japan^33^, injury risk from motor vehicle accidents in Maryland^34^, wheat production systems in India^35^, and extreme air pollution events in U.S.^36^. Additionally, temperature extremes impact soil and vegetation processes. Hatfield and Prueger^37^ showed that temperature extremes could greatly affect plant production by impacting the pollination. Crabbe *et al*.^38^ reported that extremely warm springs and autumns affect forest phenology and productivity during 2003–2011 in Europe. Changes in temperature extremes ought to affect the water cycle and the hydrological processes through altering the land-atmosphere interaction and evaporation which would in turn affect the water resources. Clearly, examining the spatiotemporal distributions of temperature extremes over the river basins in a region constitutes the first step towards understanding the relationship of temperature extremes and water cycle and a finer temporal resolution beyond annual and seasonal scales is needed for the modeling of water resources and ecosystems.

In this research work, we aim to understand the spatial and temporal distributions of monthly and annual temperature extremes over the Tibetan Plateau and its surroundings (TPS) and over its major river basins during 1963–2015 using the longest available and the most up-to-date datasets. The TP, with an average elevation of about 4000 m and an area of about 2.5 × 10^6^ km^2^, exerts profound influence on regional and global weather and climate through thermal and mechanical forcing^39–41^. The TP is also the source region of nine major Asian rivers that support 1.65 billion people and numerous ecosystems locally and downstream^42,43^. Mean temperature has been examined extensively on the TP in recent years. For example, Liu and Chen^44^ analyzed the station surface air temperature records and revealed that the main portion of the TP has experienced statistically significant warming since the mid-1950s. Wang *et al*.^45^ reported that surface air temperature on the TP increases by about 1.8 °C during 1960–2007, or 0.36 °C per decade. You *et al*.^46^ investigated temperature trends at surface stations in the eastern and central TP and showed general warming trends especially in winter at the majority of the stations. Wang *et al*.^47^ documented that during 1979–2012, annual temperature on the TP increases at 0.42 °C decade^−1^ and the winter increase is higher at 0.48 °C decade^−1^. Clearly, warming happens over the TP in recent decades and the warming rate on the TP exceeds the averages for the Northern Hemisphere and the same latitudinal zone^44^.

In comparison, temperature extremes on the TPS have not been widely studied especially in terms of regional variations and monthly distributions. A few studies that have examined temperature extremes on the TP include You *et al*.^48^ who showed that during 1961–2005 the regional occurrence of extreme cold days and nights has decreased by −0.85 and −2.38 d decade^−1^, respectively, and warm days and nights increased by 1.26 and 2.54 d decade^−1^, respectively, in the eastern and central TP; Cuo *et al*.^49^ reported that during 1957–2009 the averaged temperature minima and maxima over the northern TP increase by 0.04 and 0.03 °C year^−1^ (°Cy^−1^), respectively; Wang *et al*.^50^ revealed that during 1973–2011 most cold-related (warm-related) indices of temperature extremes show decreases (increases) over the western TP. These studies focused on the sub-regions of the TP but it is difficult to infer regional variations of temperature extremes from these studies due to the different time periods and different datasets used. Also, studies on the monthly variations of temperature extremes are lacking.

Temperature changes over the major river basins on the TPS have received a lot of attention due to their potential impacts on the hydrological processes and water resources. Cuo *et al*.^49^ showed that annual mean temperature over the upper Yellow River Basin rises by ~0.30 °C decade^−1^ during 1957–2009. Tang *et al*.^51^ presented an annual temperature trend of 0.30 °C decades^−1^ during 1961–2010 in the Tianshan Mountains river basin. Qin *et al*.^52^ examined the reconstructed surface air temperature in the western Qilian Mountains (WQM) river basin during 1957–2013 and revealed significant warming trends, indicating the shrinkage of and reduced contributions from glaciers in the region. Wang *et al*.^53^ noted a systematic increase of freezing level heights over most glacierized areas of High Asia including the WQM. Meng *et al*.^54^ reported a significant warming trend of ~0.35 °C decade^−1^ during 1961–2013 over the source region of the Yellow River basin (YLR). Wang *et al*.^55^ and Jiang *et al*.^56^ found warming trends in the range of 0.06–0.30 °Cy^−1^ during recent decades in the headwaters of the Yangtze River Basin (YTR). It is less clear, however, how temperature extremes distribute and change in the major river basins across the TPS as few studies have focused on the extremes.

The structure of this paper is as follows. Section 2 describes the study domain, datasets and methodology. The analysis results are presented in Section 3. Section 4 contains the discussions and Section 5 lists the major conclusions.

## Study Domain, Data and Methodology

### Study domain

**Figure 1:**
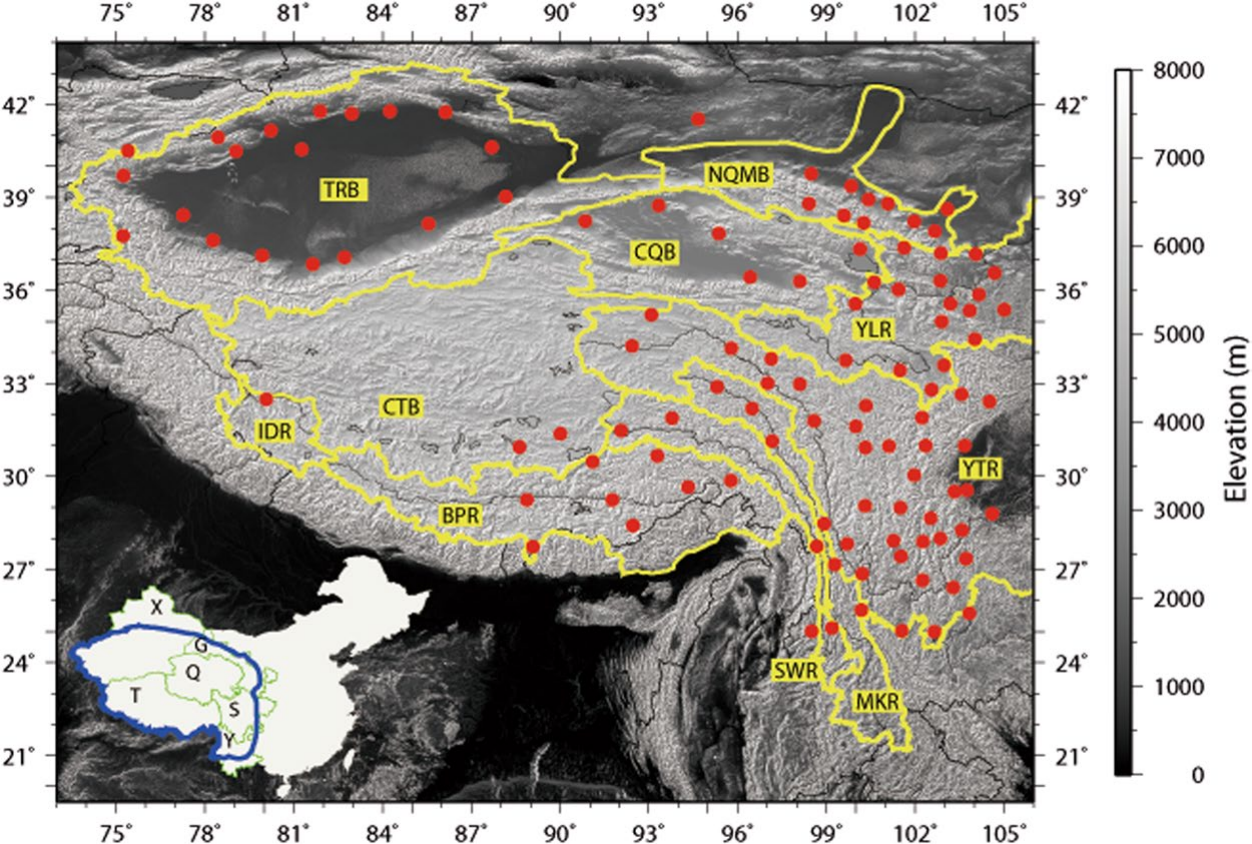
The geographic locations of the study area and the weather stations on the Tibetan Plateau and the surroundings (TPS) used in the study. Blue line in the inset represents the study area and green lines represent the boundaries of the 6 provinces including the Tibet Autonomous Region (T), Qinghai Province (Q), Xinjiang Uygur Autonomous Region (X), Gansu Province (G), Sichuan Province (S), and Yunnan Province (Y). Red points denote the weather stations. Yellow lines represent the boundaries of the ten basins on the TPS: the Yellow River basin (YLR), the Yangtze River basin (YTR), the Mekong River basin (MKR), the Salween River basin (SWR), the Brahmaputra river basin (BPR), the Indus River basin (IDR), the Tarim River basin (TRB), the Northern Qilian Mountain river basin (NQMB), the Changtang Basin (CTB), and the Chaidamu and Qinghai Lake basins (CQB) in the interior TPS. This figure was plotted using the Generic Mapping Tools (GMT) V4.5.0 (https://www.soest. hawaii.edu/gmt/).

The TPS (23°–43°N, 73°–106°E) includes the Tibet Autonomous Region (T), Qinghai Province (Q), southern Xinjiang Uygur Autonomous Region (X), part of Gansu Province (G), western Sichuan Province (S), and northern Yunnan Province (Y) (see inset in Fig. 1). The TPS is characterized by complex terrain and several mountain ranges with elevations ranging from 500 m to more than 8000 m above sea level. The Hengduan Mountain range, located in the southeastern TPS, strides 13 latitudes, encompasses 6 climatic zones, and features complex landform and geological structures^57^ (Fig. 1). The Himalaya Mountain range is the southern arch of the TPS. In the western TPS, neighboring the Pamir Plateau, the Karakoram and the west Kunlun Mountain ranges stand. The Qilian Mountain range situates in the northern TPS and separates the TP from the lowland deserts. In the warm season (May - September), the southern TPS is predominantly influenced by the south Asia monsoon, the eastern TPS is affected by the east and south Asia monsoons, and the northwest is dominated by the westerlies. The entire region is controlled by the westerlies throughout the cold season (October - April).

The TPS is of strategic importance in water resources because it is the headwater of several large Asian rivers (Fig. 1) and it is hence dubbed the “Asian Water Tower”. The large rivers include the Yellow River (YLR) and the Yangtze River (YTR) in the eastern TPS; the Mekong River (MKR) and the Salween River (SWR) in the southeast; the Brahmaputra River (BPR) in the south and the Indus River (IDR) in the southwest; as well as China’s largest interior basin, the Tarim River basin (TRB) in the northwest. There are also a few relatively smaller rivers that support the local societies and ecosystems. Among them, the Northern Qilian Mountain river basin (NQMB) is composed of several smaller rivers including the Hei River, Shiyang River, Beida River, and Shule River that all flow to the desert lowland north of the TP. The Changtang Basin (CTB), the Chaidamu and Qinghai Lake basins (CQB) are interior river basins located in the heartland of the TP. All these rivers are fed by precipitation, melt water and groundwater^42^.

### Data

Observed daily temperature maxima and minima at the weather stations were obtained from the Climate Data Center of China Meteorological Administration (CMA; http://cdc.cma.gov.cn/home.do). First of all, the stations were chosen based primarily on the location and the length of valid record. Then the data quality was checked at each station and unqualified stations were discarded. To ensure the data quality, the daily, monthly and annual time series of temperature maxima and minima were plotted for each station and visually inspected for any missing and abrupt shifts before the subsequent quality check was applied. The missing daily values were filled in based on the procedures described in the next paragraph. Afterwards, the monthly and annual time series were plotted and visually checked again. Any stations that displayed dubious monthly and annual changes and abrupt shifts were discarded further. We believe that the rigorous quality check processes employed at daily, monthly and annual time steps at each station guaranteed the data quality as well as the representativeness of the climate conditions of the stations in the region. In fact, part of the stations with similar quality check processes applied were used in a previous study by Cuo *et al*.^49^. Here, we extended the time period to 2015 and enlarged the study area to include the entire TPS region. No other homogenization procedure was applied to the stations.

The quality of the data was controlled through the following steps. The first step was to identify the missing values and remove any abnormal values that are outside of ±60 °C, a plausible range of daily temperature extremes in the region^58^. The second step was to fill the missing values if the days with missing values were less than 5 consecutively through averaging the values 2 days before and after the missing days. If the missing period was longer than 5 days consecutively in a month, the missing values were left unchanged. Further, if the missing period was longer than one month or 21 days in a month then the entire record was not used. After the quality control, we were left with 112 stations in 1963–2015 with good temperature records for analysis. A detailed description of these 112 stations is given in the Supplementary Table S1. Figure 1 shows the locations of these stations.

The derived extreme temperature indices are based on the recommendation by the ETCCDI (Expert Team on Climate Change Detection and Indices, http://etccdi.pacificclimate.org/list_27_indices.shtml). We selected 10 indices for better representativeness and for providing a broader knowledge of extreme temperature change patterns on the TPS. The definitions of the indices are as follows:

1. Monthly minimum/maximum temperatures (monthly T_min_/T_max_) are the averages of daily minimum/maximum temperatures in a month.
2. Monthly extreme minimum/maximum temperatures (monthly extreme T_min_/T_max_) are the lowest minimum/highest maximum values of daily minimum/maximum temperatures in a month.
3. Monthly highest minimum and lowest maximum temperatures (monthly highest T_min_/lowest T_max_) are the highest daily minimum temperatures and the lowest maximum temperatures in a month, respectively.
4. Monthly frost days (FD): monthly count of days when T_min_ < 0 °C. Let T_min_^i,j^ be daily minimum temperature on day i in month j. Count the number of days where:
  $${T}_{min}^{i,j} < 0\,^\circ {\rm{C}}$$
5. Monthly icing days (ID): monthly count of days when T_max_ < 0 °C. Let T_max_^i,j^ be daily maximum temperature on day i in month j. Count the number of days where:
  $${T}_{max}^{i,j} < 0\,^\circ {\rm{C}}$$
6. Monthly summer days (SU): monthly count of days when T_max_ > 25 °C. Count the number of days where:
  $${T}_{max}^{i,j} > 25\,^\circ {\rm{C}}$$
7. Monthly tropical nights (TR): monthly count of days when T_min_ > 20 °C. Count the number of days where:

$${T}_{min}^{i,j} > 20\,^\circ {\rm{C}}$$

Annual values are calculated in the same way as the monthly values except that the time interval is a year. The analyses of extreme T_min_/T_max_ and the highest T_min_/lowest T_max_ could reveal the amplitudes of the variabilities of temperature minima and maxima across the TPS in the examined time periods. Similarly, the investigation of the extreme and mean T_min_ and T_max_ could uncover the extreme and mean temperature variabilities on the TPS in the examined time periods. These variabilities are important ecosystem indices as the ranges of temperature variations and their changes determine bioclimatic conditions that affect the plant functional types and their distributions on the TPS. The indices of FD, ID, SU and TR generally reveal the patterns and changes in cold wave and heat wave.

### Method for Trends

The Mann-Kendall test is a non-parametric test, and it has been widely used in hydrological and meteorological research^49,58–61^. According to the Mann-Kendall test^62,63^, two hypotheses (H_0_ and H_1_) are tested for time series x_t_ = (x_1_, x_2_, …, x_n_). The null hypothesis H_0_ states that x_t_ is an independently distributed random sample which means that there is no significant trend. The alternative hypothesis H_1_ states that x_t_ has a monotonically decreasing or increasing trend. The test statistic S, which has mean zero and a variance computed by Eq. (3), is calculated using Eqs (1 and 2), and is asymptotically normal:

1 $$S=\sum _{{\rm{i}}=1}^{n-1}\sum _{j=i+1}^{n}{\rm{sgn}}({x}_{j}-{x}_{i})$$

2 $${\rm{sgn}}({x}_{j}-{x}_{i})=\{\begin{array}{c}\begin{array}{cc}1, & \,\,if({x}_{j}-{x}_{i}) > 0\end{array}\\ \begin{array}{cc}0, & \,if({x}_{j}-{x}_{i})=0\end{array}\\ \begin{array}{cc}-1, & if({x}_{j}-{x}_{i}) < 0\end{array}\end{array}$$

3 $$Var(S)=\frac{n(n-1)(2n+5)-\sum _{i=1}^{m}{t}_{i}({t}_{i}-1)(2{t}_{i}+5)}{18}$$

where n is the number of data points, m is the number of tied groups, and t_i_ is the number of observations in the *i*th tied group. In cases where the sample size n > 10, the standard normal variable Z is computed by using Eq. (4).

4 $$Z=\{\begin{array}{ccc}\frac{S-1}{\sqrt{Var(S)}}, & if & S > 0\\ 0, & if & S=0\\ \frac{S+1}{\sqrt{Var(S)}}, & if & S < 0\end{array}$$

Positive (negative) values of Z indicate increasing (decreasing) trends. With two-tailed test, at a given significance level α, the null hypothesis H_0_ is rejected for an absolute value of |Z| ≥ Z_1−α/2_. In this work, the significance level is set at α = 0.05.

The non-parametric robust estimate of the magnitude of the slope, β, of linear trend, can be obtained using the method of Sen^64^ as follows:

5 $$\beta =Median[\frac{{x}_{i}-{x}_{j}}{i-j}]\,for\,all\,j < i$$

The Mann-Kendall analysis was applied to all monthly and annual variables described before to obtain the trends and their significance during 1963–2015.

## Results

### Monthly and annual means

**Figure 2:**
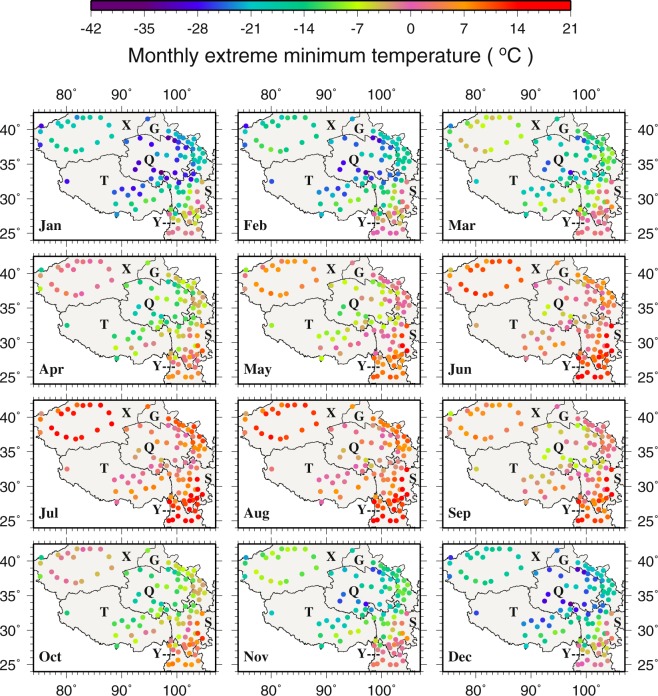
The spatial distributions of monthly extreme minimum temperature (°C). This figure was plotted using the Generic Mapping Tools (GMT) V4.5.0 (https://www.soest. hawaii.edu/gmt/).

Monthly extreme T_min_ ranges from −36.0 to 20.3 °C (Fig. 2), with the lowest and highest extreme T_min_ occurring at Qingshuihe (56034 in Q) and Yibin (56492 in S), respectively. Similar to monthly T_min_, the lowest (highest) extreme T_min_ is found in January (July and August) on the TPS. Spatially, stations in Q are associated with the lowest extreme T_min_ while stations in the southeast correspond to the highest extreme T_min_. In the coldest January, extreme T_min_ differences between the southeast and the northwest could reach more than 30.0 °C, whereas the differences are only about one third of that in summer. Even in the warmest July and August, stations in the central TPS with elevations in 2770–4672 m still show about 0.0 °C monthly extreme T_min_; in contrast, stations in the southeast display monthly extreme T_min_ ≥ 0.0 °C throughout the year. The northwest is an extreme area on the TPS in the sense that the monthly extreme T_min_ difference between the coldest January and the warmest July could reach nearly 50.0 °C. Clearly, high latitudes, dominance by the water vapor depleted westerlies, dry continental climate, and sparse vegetation coverage in the northwest all contribute to the extreme conditions there. Monthly T_min_ and the highest T_min_ spatial patterns generally follow the elevation and have similar pattern to the extreme T_min_ (see details in supplementary material and Figs S2 and S3).

**Figure 3:**
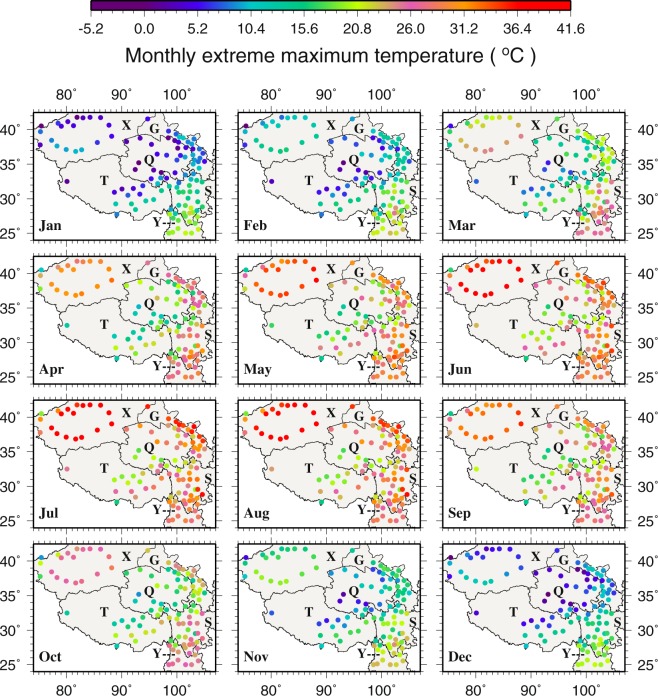
The spatial distributions of monthly extreme maximum temperature (°C). This figure was plotted using the Generic Mapping Tools (GMT) V4.5.0 (https://www.soest. hawaii.edu/gmt/).

For monthly extreme T_max_, the range is from −1.8 to 41.0 °C across the TPS (Fig. 3). The hottest area in May - September belongs to the northwest (X) where monthly extreme T_max_ reaches 41.0 °C at Ruoqiang (51777). Interestingly, the lowest monthly extreme T_max_ is also found in the northwest in January and December. This reinforces the earlier argument that the northwest is an extreme region on the TPS in the sense that it features the largest seasonal temperature variations. Comparing monthly extreme T_min_ and T_max_ at the same stations, it is found that the extreme ranges of monthly temperatures are approximately −30.0 to 30.0 °C, −32.0 to 41.0 °C, and 0.0 to 35.0 °C in the central, northwestern and southeastern TPS, respectively. Monthly T_max_ and the lowest T_max_ also have similar spatiotemporal patterns to the extreme T_max_ (see details in supplementary material and Figs S3 and S4)

Comparing the extreme and highest monthly T_min_ with the extreme and lowest monthly T_max_ at the same stations across the TPS reveals that the amplitude of T_max_ variability (~20.0 °C) is much smaller than that of T_min_ (~50.0 °C) in the northwest. In the central TPS, however, the amplitudes of T_min_ and T_max_ variability stay around the same at ~30.0 °C.

**Figure 4:**
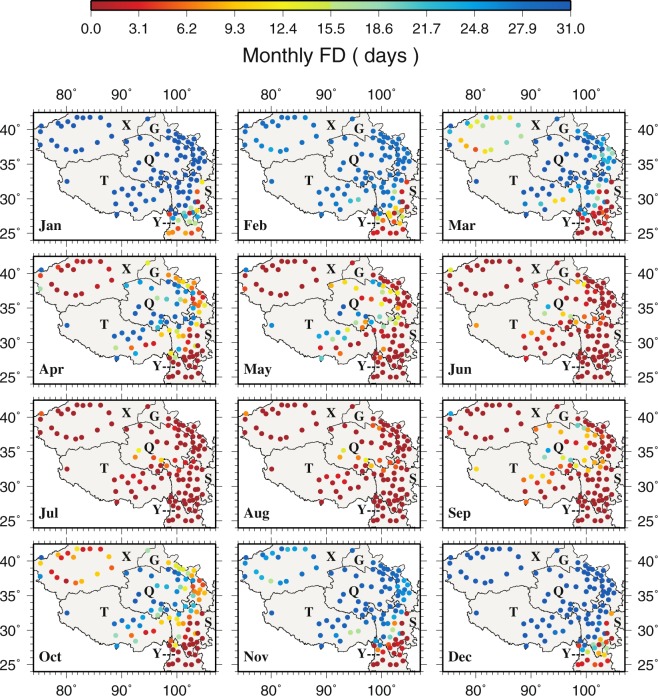
The spatial distributions of monthly frost days. This figure was plotted using the Generic Mapping Tools (GMT) V4.5.0 (https://www.soest. hawaii.edu/gmt/).

**Figure 5:**
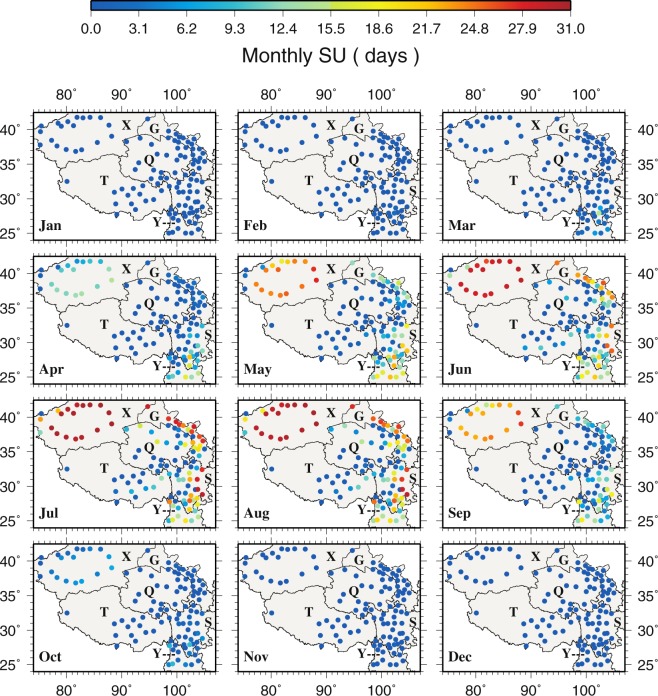
The spatial distributions of monthly summer days. This figure was plotted using the Generic Mapping Tools (GMT) V4.5.0 (https://www.soest. hawaii.edu/gmt/).

The spatial patterns of extreme cold events (FD and ID, Figs 4 and S5) and extreme warm events (SU and TR, Figs 5 and S6) correspond to extreme temperatures very well. The central TPS (Q and T) where extreme temperatures are low shows more cold events but few warm events. FD and ID are low in the southeastern TPS all year around where extreme temperatures are high. The most warm events occur in the southeastern TPS, especially at Yibin (56492) and Leshan (56386) in the eastern S. Regions (e.g., X) with greater monthly temperature differences also display greater differences in cold and warm events. Comparing to the other stations in X, Tuergate (51701) has more cold events. During January, February, November and December, monthly T_min_ in X, G, Q and T is less than 0 °C resulting in the highest occurrence of FD in the same period. From May to September, none of the stations have monthly T_max_ less than 0 °C, hence, ID is always zero during the same period over the TPS. The largest SU occurs in X during July and August, and in the northern G during July. During June to August, Yibin, Leshan and Dujiangyan (56188) in S exhibit the highest TR.

**Figure 6:**
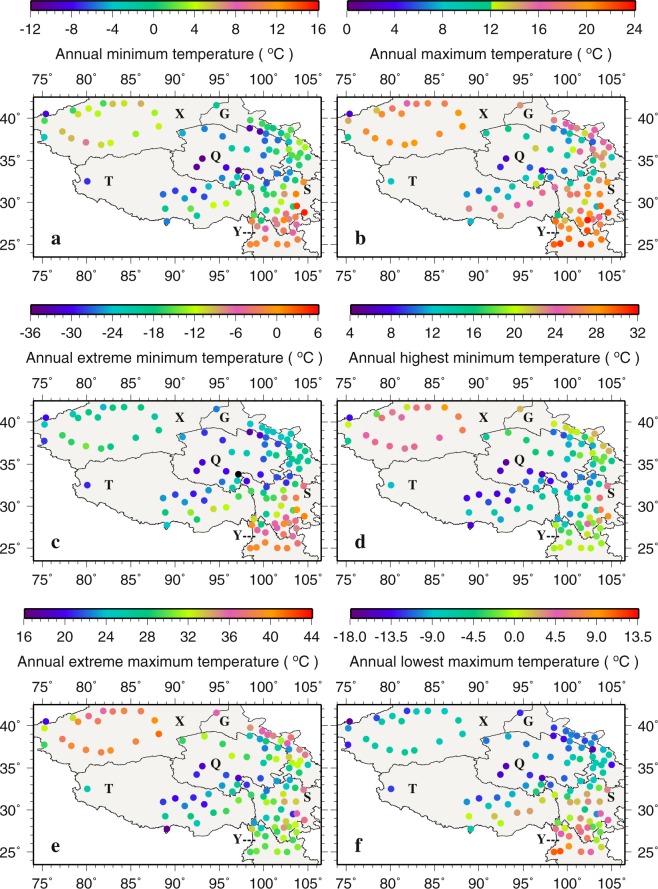
The spatial distributions of annual temperature variables (°C). This figure was plotted using the Generic Mapping Tools (GMT) V4.5.0 (https://www.soest. hawaii.edu/gmt/).

Figure 6 presents the spatial patterns of the derived annual temperature variables. Annual T_min_ ranges from −11.2 °C at Qingshuihe (56034 in Q) to 15.4 °C at Yibin (56492 in S) (Fig. 6a), and annual T_max_ ranges from 2.5 °C at Wudaoliang (52908 in Q) to 23.5 °C at Xichang (56571 in S) (Fig. 6b). Both annual T_min_ and T_max_ suggest that the central TPS is the coldest area while the southeastern TPS is the warmest area in the region, largely consistent with the spatial patterns of monthly T_min_ and T_max_. Also, consistent with monthly spatial patterns, summer T_max_ is the highest in the northwest. Differences between annual T_max_ and T_min_ at the same stations are in the range of ~15 °C in the central and northwestern TPS and ~10.0 °C in the southeastern TPS.

Annual extreme T_min_ ranges from −36.6 °C at Qingshuihe (56034 in Q) to 1.0 °C at Yibin (56492 in S) (Fig. 6c). Annual highest T_min_ is all positive across the TPS and ranges from 5.4 °C at Wudaoliang (52908 in Q) to 28.4 °C at Luerle (51656 in X) (Fig. 6d). Extreme T_min_ lower than −30.0 °C are found at Qingshuihe, Tuole (52633), Yeniugou (52645) and Tuotuohe (56004) all located in Q. Variabilities of annual T_min_, i.e., differences between annual highest and extreme T_min_, are in the range of −15.0 to 5.5 °C, −33.0 to −20.0 °C, and −33.0 to −15.0 °C for the southeastern, central and northwestern TPS, respectively, further indicating that the central TPS is the coldest area followed by the northwest in the region.

Annual extreme T_max_ is positive across the TPS and ranges from 16.7 °C at Pali (55773 in T) to 41.0 °C at Ruoqiang (51777 in X) (Fig. 6e), and annual lowest T_max_ ranges from −17.0 °C at Tuergate (51701 in X) to 12.3 °C at Baoshan (56748 in Y) (Fig. 6f). The lowest annual extreme T_max_ of 17.0–25.0 °C are seen in the central TPS (Q and T). Around the periphery of the central TPS, annual extreme T_max_ gradually declines from the northwest (mostly in 30.0 to 41.0 °C) to the northeast (30.0 to 37.0 °C) and to the southeast (25.0 to 35.0 °C). Annual lowest T_max_ in X, G, Q, the central T and the northwestern S, ranging from −17.0 to −5.0 °C, does not display large spatial variations. Annual lowest T_max_ is all above 0.0 °C in Y.

**Figure 7:**
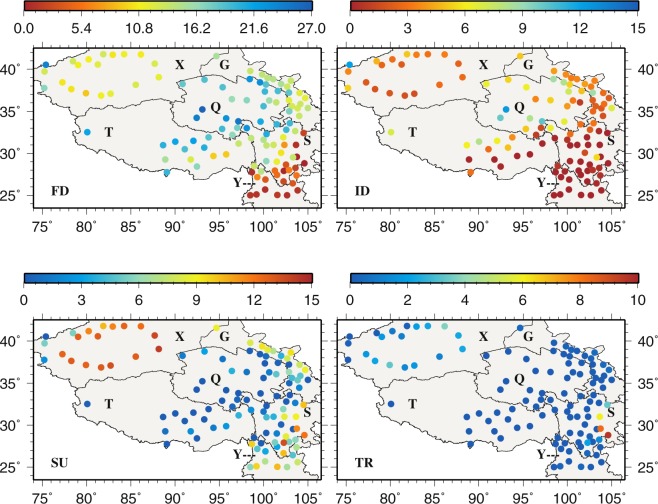
The spatial distributions of annual frost days, icing days, summer days, and tropical night days. This figure was plotted using the Generic Mapping Tools (GMT) V4.5.0 (https://www.soest. hawaii.edu/gmt/).

Annually, FD (TR) occurs most (least) often at the same stations across the region (Fig. 7). The largest and smallest annual FDs are noted in the central (Q) and the southeastern (Y) TPS, respectively. The spatial patterns of annual ID resemble those of annual FD to a large extent, possibly indicating that cold days are followed by cold nights and vise versa. In contrast, SU and TR do not seem to bear strong similarity in spatial patterns. For SU, most of X and the border between S and Y display the highest annual number, whereas the least number is found in Q and T. Annual TR is generally small across the TPS except in the eastern S (e.g., Yibin, 56492, 9.5 days; Leshan, 56386, 8.2 days; Dujiangyan, 56188, 5.9 days) and most of X. In general, warm events frequent at Yibin, Leshan and Dujiangyan in S while cold events often occur at Wudaoliang (52908), Qingshuihe (56034) and Tuotuohe (56004) in Q and Tuergate (51701) in X.

Based on the distinct characteristics of the monthly and annual temperature variables (except TR which is small) on the TPS, four sub-regions, i.e., the center, the northeast, the northwest and the southeast are identified. The spatial variations of temperatures are determined by elevation, latitudes, land cover and atmospheric circulations, consistent with what has been reported by Yu *et al*.^65^, Wang X *et al*.^66^ and Su *et al*.^67^. In this region, the coldest area is the central TPS due to the high elevation, and the warmest area is the southeastern TPS in the annual sense due to the low elevation, low latitudes and monsoonal climate. The northwestern TPS is the hottest in the summer months. The northeastern TPS appears to stay in between, neither too hot nor too cold, except for a few stations along the ridge of the Qilian Mountain Range where T_max_ and T_min_ are much lower than the surrounding stations. The largest monthly temperature variations occur in the northwest (X) that lies in the northernmost latitudes of the TPS, has sparse vegetation, and is also controlled by the dry westerlies.

### Monthly and annual trends

**Figure 8:**
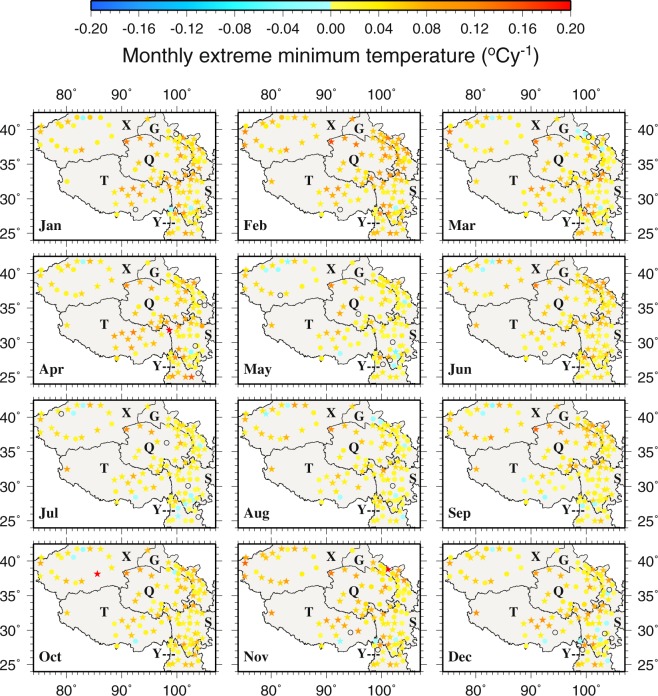
The spatial distributions of the trends of monthly extreme minimum temperature during 1963–2015. Black circles represent zero trends. Stars represent statistically significant trends (*p* < 0.05). This figure was plotted using the Generic Mapping Tools (GMT) V4.5.0 (https://www.soest. hawaii.edu/gmt/).

**Figure 9:**
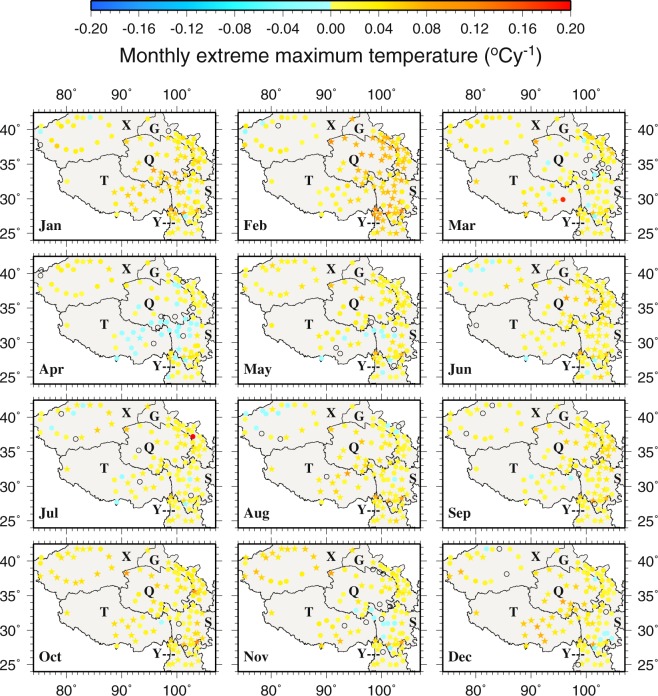
The spatial distributions of the trends of monthly extreme maximum temperature during 1963–2015. Black circles represent zero trends. Stars represent statistically significant trends (*p* < 0.05). This figure was plotted using the Generic Mapping Tools (GMT) V4.5.0 (https://www.soest. hawaii.edu/gmt/).

**Figure 10:**
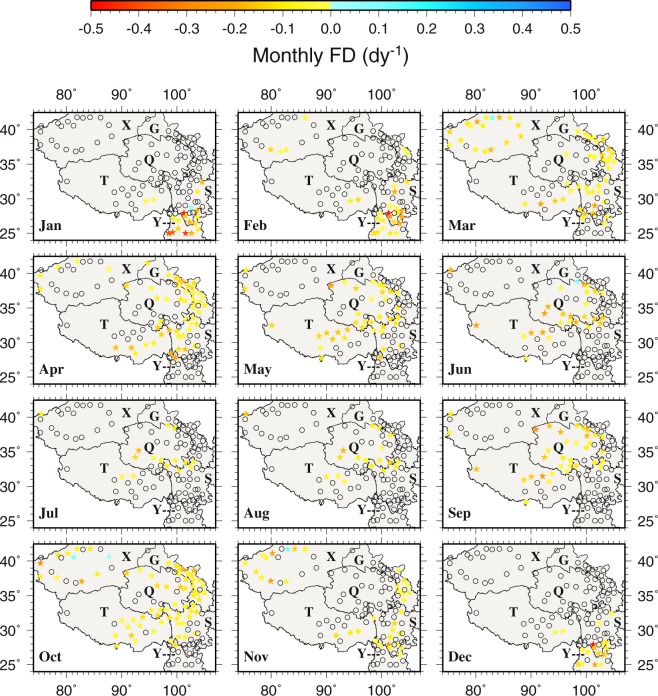
The spatial distributions of the trends of monthly frost days during 1963–2015. Black circles represent zero trends. Stars represent statistically significant trends (p < 0.05). This figure was plotted using the Generic Mapping Tools (GMT) V4.5.0 (https://www.soest. hawaii.edu/gmt/).

**Figure 11:**
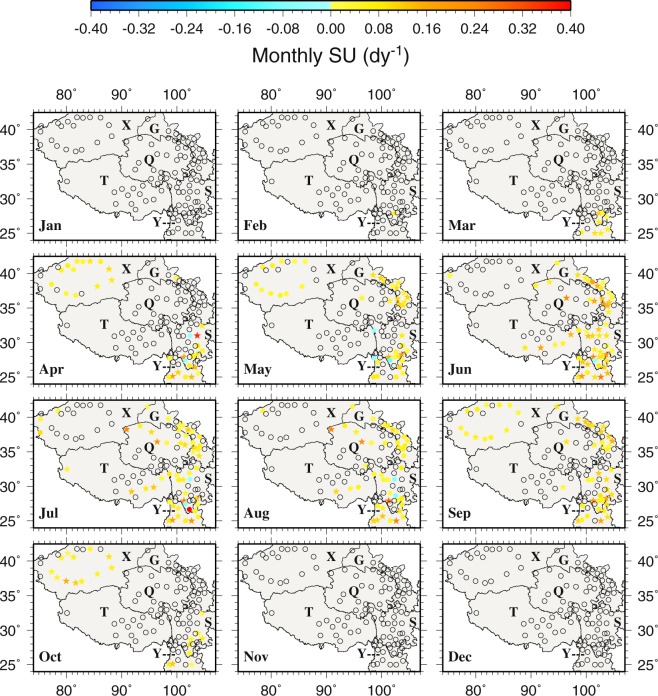
The spatial distributions of the trends of monthly summer days during 1963–2015. Black circles represent zero trends. Stars represent statistically significant trends (p < 0.05). This figure was plotted using the Generic Mapping Tools (GMT) V4.5.0 (https://www.soest. hawaii.edu/gmt/).

**Figure 12:**
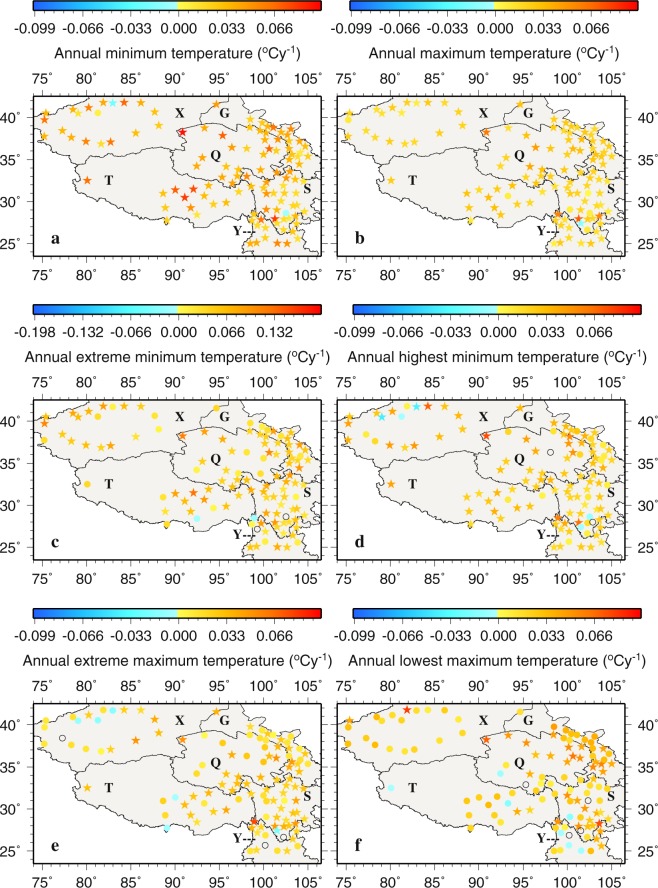
The spatial distributions of the trends of annual temperature variables during 1963–2015. Black circles represent zero trends. Stars represent statistically significant trends (*p* < 0.05). This figure was plotted using the Generic Mapping Tools (GMT) V4.5.0 (https://www.soest. hawaii.edu/gmt/).

**Figure 13:**
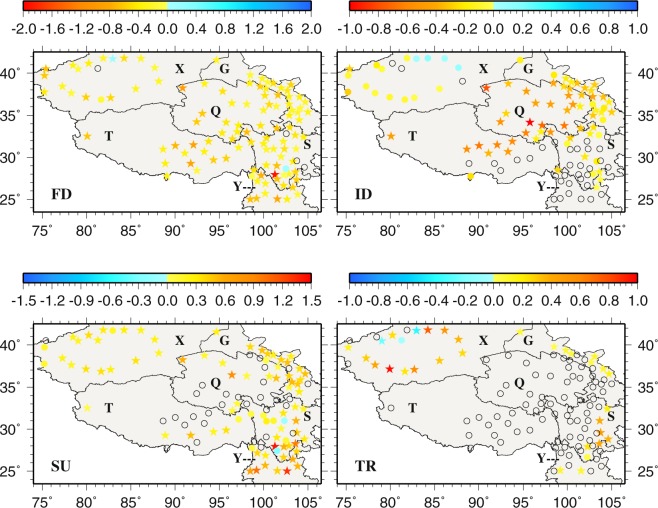
The spatial distributions of the trends of annual frost days, icing days, summer days and tropical nights (dy^−1^) during 1963–2015. Blocks represent zero trends Stars represent statistically significant trends (p < 0.05). This figure was plotted using the Generic Mapping Tools (GMT) V4.5.0 (https://www.soest. hawaii.edu/gmt/).

Monthly trends of the six temperature variables (units: °C year^−1^ abbreviated as °Cy^−1^) and four extreme climate indices (units: days year^−1^ bbreviated as dy^−1^) in 1963–2015 are shown in Figs 8–11 and S7–S11 in supplementary, with the statistically significant trends at *p* < 0.05 represented by stars. The trends of the six annual temperature variables (in °Cy^−1^) and four extreme climate indices (units: days year^−1^ abbreviated as dy^−1^) are presented in Figs 12 and 13.

The trends of monthly extreme T_min_ on the TPS are positive at most stations (i.e., 93% of stations in July, 100% in February and other months in between) (Fig. 8). The trends range from −0.0500 °Cy^−1^ at Yuexi (56475 in S) in May to 0.2000 °Cy^−1^ at Qiemo (51855 in X) in October. For February, the increasing trends at 81% of the stations are statistically significant. The statistically significant decreasing trends tend to be located along the periphery of the central TPS and mainly in the warm season, e.g., −0.0396 °Cy^−1^ at Kuche (51644 in the northwest) in May, −0.0500 °Cy^−1^ at Yuexi (56475 in the eastern S) in May, −0.0179 °Cy^−1^ at Yanyuan (56565 in the eastern S) in July, −0.0163 °Cy^−1^ at Dali (56751 in the southern Y) in July, and −0.0343 °Cy^−1^ at Longzi (55696 in the southern T) in November. Monthly extreme T_min_ increases more in the central TPS and the northwest than the other areas. The positive trends at more than half of the stations in T are statistically significant. As for monthly T_min_, amongst the months, February features all positive and also the largest trends, with 94% of the stations showing statistically significant trends (Fig. S7). Similar to monthly T_min_ and monthly extreme T_min_, monthly highest T_min_ also displays predominantly increasing trends across the TPS and throughout the year, with normally less than 10 stations showing decreasing trends during any month (Fig. S8).

The trends of monthly extreme T_max_ range from −0.0400 °Cy^−1^ at Jiali (56202 in T) in April to 0.1800 °Cy^−1^ at Bomi (56227 in T) in March (Fig. 9). The trends of monthly extreme T_max_ are mostly positive on the TPS throughout the year and 63%, 59% and 55% of the stations correspond to statistically significant positive trends in February, June and October, respectively. April is an exception in the sense that for this month about 30% of the stations that are located in the central T and near the borders of T, Q and S show decreasing trends although statistically significant only at Jiali (56202 in T, −0.0400 °Cy^−1^), Daocheng (56357 in S, −0.0250 °Cy^−1^) and Yanyuan (56565 in S, −0.0281 °Cy^−1^). It is worth noting that only Yanyuan (56565) in S displays statistically significant negative trends, ranging from −0.0346 to −0.0194 °Cy^−1^, in several months including March, May, June and December. Also, September sees the smallest increasing trends amongst all months. Spatially, the eastern T in January and the eastern TPS in February show large and statistically significant increasing trends. The decreasing trends of monthly T_max_, located mainly along the borders of T, X, Q and S, are noted primarily in April (Fig. S9). Monthly lowest T_max_ exhibits the largest and the most number of statistically significant increasing trends in February near the borders of S and Y as well as in the central Y; while the decreasing trends are found in April, July and August in the eastern TPS and in December and January in the northwestern TPS (Fig. S10).

The monthly FD trends are rather small over most of the TPS (Fig. 10). Statistically significant negative trends are found mainly in the southern Y and the border between Y and S, especially at Muli (56459 in S), from December to February. Only Kuche (51644 in X, 0.1250 dy^−1^ in November and 0.0833 dy^−1^ in March), Alaer (51730 in X, 0.0952 dy^−1^ in October), Yuexi (56475 in S, 0.0588 dy^−1^ in January) and Tieganlike (51765 in X, 0.0238 dy^−1^ in October) show positive trends but none of them are statistically significant (Fig. 10). For ID, almost all stations show zero monthly trends from April to September (Fig. S11) because of zero occurrence in the warm months (Fig. S5).

Most stations show zero monthly SU trends in all 12 months (Fig. 11) because of few occurrences of daily maximum temperature greater than 25 °C (Fig. 5). Positive and negative trends mainly occur in the warm months from April to September. Statistically significant positive SU trends are found at Huili (56671 in S, 0.4000 dy^−1^) in April, Kunming (56778 in Y, 0.2593 dy^−1^) in June, Mangai (0.2500 dy^−1^ in July and August) and Linxia (52984 in G, 0.1892 dy^−1^). Statistically significant negative SU trends are noted at Xiaojin (56178, −0.0682 dy^−1^ in August) and Yanyuan (56565, −0.1034 dy^−1^ in May, −0.0500 dy^−1^ in April and −0.0435 dy^−1^ in July) in S. Similar to SU, most stations in all 12 months also show zero monthly TR trends because of low minimum temperature in general in the region (Fig. S12).

Annual temperature variables except annual lowest T_max_ show predominantly positive trends, and the positive trends are statistically significant at 97%, 94%, 75%, 78%, and 58% of the stations for annual T_min_, annual T_max_, annual extreme T_min_, annual highest T_min_, and annual extreme T_max_, respectively (Fig. 12). Statistically significant negative trends are only found at Kuche (51644 in X, −0.0240 °Cy^−1^) for annual T_min_, Yanyuan (56565 in S, −0.0150 °Cy^−1^) for annual T_max_, Kuche (−0.0405 °Cy^−1^) and Keping (51720 in X, −0.0430 °Cy^−1^) for annual highest T_min_, and Yanyuan (−0.0214 °Cy^−1^) for annual extreme T_max_. For both annual extreme T_min_ and lowest T_max_, there are a few stations that show negative trends but none of them are statistically significant.

The largest positive trends are noted at Mangai (51886 in Q, 0.1000 °Cy^−1^) for annual T_min_, Muli (56459 in S, 0.0170 °Cy^−1^) for annual T_max_, Naqu (55299 in T, 0.1300 °Cy^−1^) for annual extreme T_min_, Mangai (51886 in Q, 0.0760 °Cy^−1^) for annual highest T_min_, Deqin (56444 in Y, 0.0770 °Cy^−1^) for annual extreme T_max_, and Baicheng (51633 in X, 0.080 °Cy^−1^) for annual lowest T_max_ (Fig. 12). Clearly, the magnitudes of the largest trends for the T_min_ variables are generally higher than those for the T_max_ variables. This also holds true for the monthly variables.

The spatial patterns of the trends of annual temperature variables largely follow those of the same monthly temperature variables. For the annual T_min_ variables, the central, northwestern and eastern TPS are where large increasing trends are normally located (Fig. 12a,c,d) while for the annual T_max_ variables, large increasing trends tend to be found more often in the northern and central Q than elsewhere (Fig. 12b,e,f).

In summary, about 87% and 71% of stations show positive trends for monthly T_min_ and T_max_, respectively. Notably, February is when most positive trends for both T_min_ and T_max_ are found among all the months, and 81% (41%) of the positive trends for T_min_ (T_max_) are statistically significant. For the monthly T_min_ variables, statistically significant negative trends mainly occur in May and July and are also concentrated in the northwest and near the borders of T, S and Y. Kuche (51644) is the station that often corresponds to the statistically significant decreasing trends for T_min_. For the monthly T_max_ variables, negative trends tend to be located in S, and Yanyuan (56565) is the station where statistically significant negative trends are frequently observed.

For annual and extreme T_min_, 96% of stations display positive trends, among which 97% and 75% are statistically significant, respectively. Negative trends tend to be located in the central X, especially at Kuche, similar to the monthly T_min_ variables. A few non-significant decreasing trends of annual T_min_ also appear in the southern T and the central S. For annual and extreme T_max_, 95% and 99% of stations exhibit positive trends, out of which 94% and 58% are statistically significant, respectively. A few negative trends for T_max_ are noted in the central X and near the borders of T, Y and S. Similar to the monthly T_max_ variables, the largest negative trends are observed at Yanyuan for the annual T_max_ variables.

Annually (Fig. 13), only Kuche (0.1935 dy^−1^, significant) in X and Yuexi (0.0909 dy^−1^) in S show positive FD trends. The most significant negative FD trend occurs at Muli (−1.9189 dy^−1^) in S. The central TPS corresponds to statistically significant negative trends for both annual FD and ID. The zero trends of annual ID (35 stations) are mostly seen in Y and S, the southern T and the northeastern X. Luntai (0.1429 dy^−1^), Tieganlike (0.0909 dy^−1^), Kuche (0.0476 dy^−1^) and Luerle (0.0455 dy^−1^) in X show statistically significant positive ID trends. For SU, only Yanyuan (−0.2500 dy^−1^) and Xiaojin (−0.0857 dy^−1^) in S display negative trends, but not statistically significant. Statistically significant positive SU trends occur in Y and in the border between Y and S. As for TR, statistically significant positive and negative trends occur in X, e.g., Hetian (0.9655 dy^−1^), Luntai (0.7500 dy^−1^), Minfeng (0.5926 dy^−1^), Luerle (0.5217 dy^−1^), Kuche (−0.4167 dy^−1^) and Keping (−0.2439 dy^−1^). However, most stations (80 stations) exhibit zero annual TR trends.

**Figure 14:**
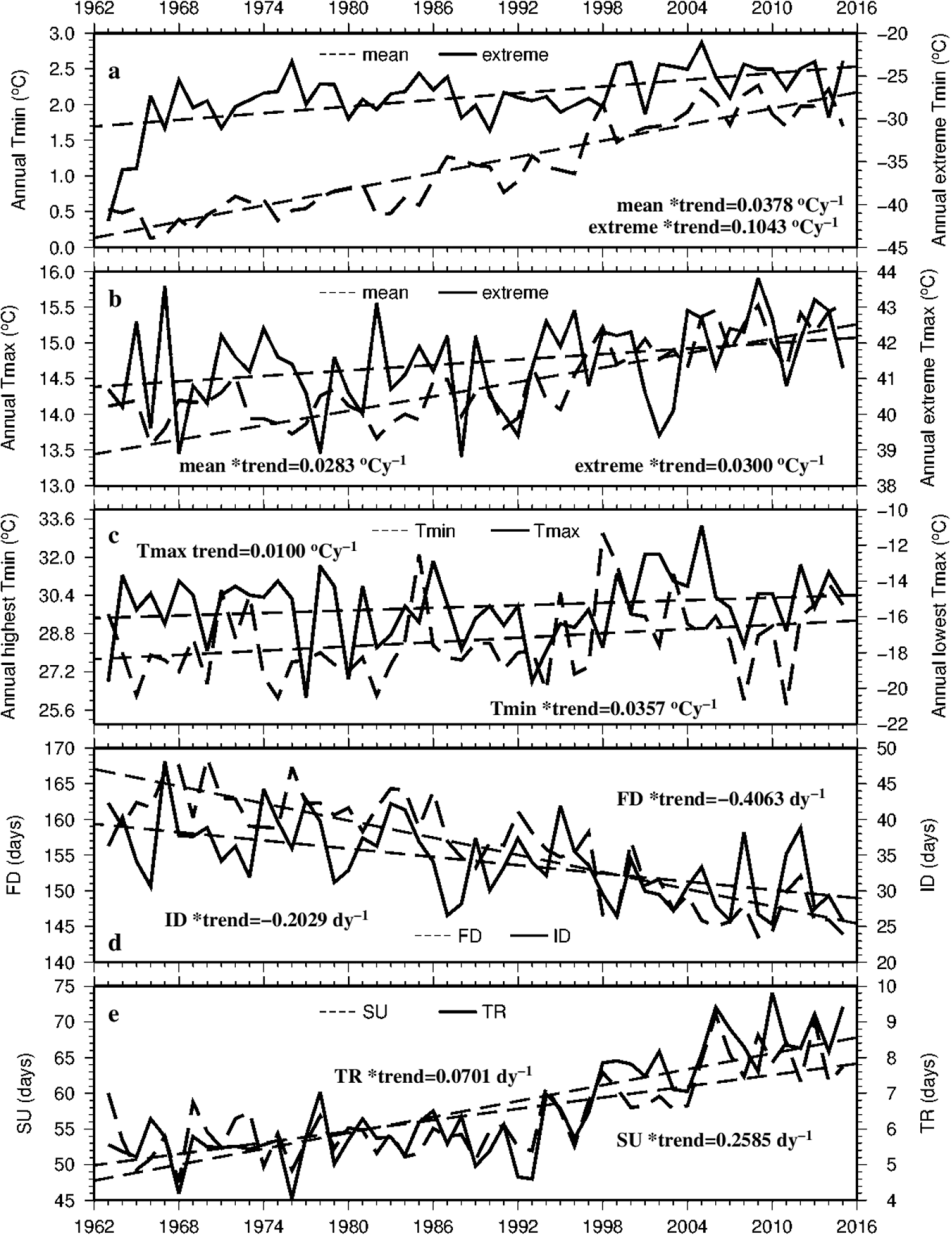
The temporal variations of regionally averaged annual extreme variables during 1963–2015. Stars represent statistically significant trends (p < 0.05). This figure was plotted using the Generic Mapping Tools (GMT) V4.5.0 (https://www.soest. hawaii.edu/gmt/).

In an effort to represent the regional mean extreme temperature conditions and changes, the 112-station averaged annual T_min_, extreme T_min_, T_max_, extreme T_max_, highest T_min_ and lowest T_max_ time series are computed and presented in Fig. 14a,b,c. The trends of annual extreme T_min_ and T_max_ variables except lowest T_max_ are all positive and statistically significant, and extreme T_min_ variables display larger trends than extreme T_max_ variables. Regionally (112-station) averaged, annual extreme cold events (FD and ID) decrease significantly and annual extreme warm events (SU and TR) increase significantly during 1963–2015 (Fig. 14d,e). FD even decreases at 0.4 dy^−1^, the highest rate among the 4 cold and warm indices. Worthy of mentioning is that regional mean SU and TR exhibit quite similar temporal variability.

### Basin temperatures and their changes

This section examines the means and trends of monthly and annual T_min_ and T_max_ during 1963–2015 for the ten large river basins on the TPS. The geographic locations of the river basins are indicated in Fig. 1. The Supplementary Table S1 lists the stations that fall within each of the river basins. The monthly and annual temperatures of all stations located in the same basin were averaged to obtain the basin averaged monthly and annual temperatures, from which the temporal means and trends were computed for each basin. Depending on the availability of station data, the number of stations for each basin varies from one (the Indus River, IDR) to two (the Changtang Basin, CTB) to thirty-seven (the Yangtze River, YTR) (Fig. 1 and Table 1). Due to station scarcity, the means and trends for IDR and CTB were calculated based on only one and two stations, respectively; thus, caution needs to be exerted here when interpreting the results although we would argue that the means and trends might still be representative to some extent because of the subdued topography in the two basins. Our goal here is to investigate the conditions and changes of basin temperatures during the same time period using available data, as there has been lack of such information for all ten river basins on the TPS.

**Table 1:** Monthly and annual Tmin and Tmax (°C) for ten river basins.

	BPR (8)	CQB (6)	CTB (2)	IDR (1)	MKR (5)	NQMB (10)	SWR (4)	TRB (19)	YLR (17)	YTR (37)
**T**_**min**_										
Jan	−12.3	−18.6	−17.5	−20.1	−8.0	−18.3	−10.5	−13.9	−13.6	−7.0
Feb	−9.4	−15.0	−15.3	−17.3	−5.5	−14.6	−8.2	−9.0	−10.1	−4.6
Mar	−5.3	−9.3	−11.5	−13.0	−1.8	−7.7	−4.5	−1.0	−4.4	−0.7
Apr	−1.3	−3.4	−6.6	−8.1	2.0	−0.8	−0.4	6.3	0.9	3.5
May	2.9	1.9	−2.6	−3.5	6.1	4.8	4.0	11.1	5.4	7.7
Jun	7.0	6.1	2.2	2.7	10.1	9.0	8.1	14.6	8.9	10.7
Jul	8.4	8.9	4.3	7.2	11.2	11.7	9.5	16.4	11.1	12.1
Aug	7.4	7.9	3.8	7.1	10.5	10.6	9.1	15.4	10.4	11.7
Sep	5.8	3.0	1.4	1.5	8.4	5.6	6.8	10.2	7.0	9.3
Oct	−0.3	−4.4	−5.3	−8.2	3.3	−1.9	1.8	2.6	1.2	4.7
Nov	−7.0	−11.8	−12.4	−14.7	−3.2	−9.8	−4.9	−4.7	−6.0	−1.5
Dec	−11.2	−17.0	−16.3	−18.8	−12.9	−16.2	−17.8	−11.3	−13.5	−7.4
Annual	−1.2	−4.3	−6.3	−7.1	2.1	0.4	0.2	3.9	0.0	6.0
**T**_**max**_										
Jan	4.9	−3.3	−3.4	−4.3	7.5	−2.2	4.4	−1.2	1.4	7.5
Feb	6.4	0.5	−1.7	−2.4	9.0	1.6	6.0	4.0	4.2	9.5
Mar	9.4	6.1	1.9	2.0	12.4	8.0	9.4	12.8	9.3	13.4
Apr	12.8	11.9	6.3	7.4	15.9	15.0	12.8	20.7	14.6	17.2
May	16.4	16.4	10.3	12.3	19.4	20.0	16.2	25.4	18.3	19.9
Jun	19.3	19.8	15.0	18.2	21.0	23.8	18.6	29.0	21.0	21.1
Jul	19.6	22.3	15.6	21.4	22.5	25.8	19.4	30.8	22.9	22.4
Aug	18.7	21.9	14.5	20.4	22.0	24.9	19.2	29.9	22.4	22.3
Sep	17.5	17.2	12.6	16.2	20.1	20.0	17.5	25.3	18.2	19.5
Oct	13.9	10.7	6.8	8.4	16.1	13.6	13.5	18.3	13.4	15.6
Nov	9.6	3.3	1.2	2.7	11.9	5.4	8.9	8.8	7.4	11.6
Dec	6.5	−1.8	−1.8	−1.8	4.5	−0.7	−0.3	0.7	1.2	6.9
Annual	13.0	10.5	6.4	8.4	15.6	15.0	12.7	18.6	12.9	17.7

The number in the brackets represents the number of weather stations for each basin.

Tables 1 and 2 show the means and trends of monthly and annual T_min_ and T_max_ for the ten river basins. Mean monthly T_min_ ranges from −20.1 °C in January for IDR to 16.4 °C in July for TRB (Table 1). TRB, located entirely in the northwest, displays the largest monthly T_min_ variability of 30.3 °C (i.e., −13.9 °C in January and 16.4 °C in July). TRB also shows the highest T_min_ during April – September among the ten basins. YTR, whose major section is situated in the southeast, exhibits the smallest monthly T_min_ variability of 19.5 °C (i.e., −7.4 °C in December and 12.1 °C in July). IDR and CTB, located in the interior TPS, are the two coldest basins in the TPS in terms of T_min_ because of high elevation. Monthly T_min_ is negative (positive) for all basins during November – March (June – September), and April, May and October are the transition months with negative T_min_ for some basins but positive T_min_ for the other basins.

**Table 2:** The trends of monthly and annual Tmin and Tmax (°C y^−1^) for ten river basins.

	BPR	CQB	CTB	IDR	MKR	NQMB	SWR	TRB	YLR	YTR
**T**_**min**_										
Jan	0.0425*	0.0532*	0.0776*	0.0237*	0.0333*	0.0459*	0.0520*	0.0349*	0.0479*	0.0380*
Feb	0.0247*	0.0890*	0.0722*	0.0337*	0.0325*	0.0771*	0.0533*	0.0562*	0.0646*	0.0494*
Mar	0.0356*	0.0495*	0.0724*	0.0387*	0.0418*	0.0308*	0.0613*	0.0362*	0.0342*	0.0385*
Apr	0.0293*	0.0356*	0.0373*	0.0421*	0.0323*	0.0435*	0.0468*	0.0299*	0.0269*	0.0297*
May	0.0261*	0.0380*	0.0473*	0.0765*	0.0233*	0.0370*	0.0361*	0.0298*	0.0248*	0.0213*
Jun	0.0275*	0.0569*	0.0333*	0.0707*	0.0273*	0.0526*	0.0360*	0.0326*	0.0436*	0.0315*
Jul	0.0260*	0.0467*	0.0294*	0.0640*	0.0165*	0.0351*	0.0343*	0.0263*	0.0248*	0.0153*
Aug	0.0248*	0.0449*	0.0206*	0.0534*	0.0176*	0.0255*	0.0310*	0.0302*	0.0207*	0.0185*
Sep	0.0245*	0.0558*	0.0244*	0.0560*	0.0211*	0.0413*	0.0415*	0.0287*	0.0294*	0.0243*
Oct	0.0232*	0.0534*	0.0326*	0.0672*	0.0254*	0.0387*	0.0334*	0.0314*	0.0314*	0.0280*
Nov	0.0306*	0.0613*	0.0709*	0.0939*	0.0332*	0.0430*	0.0513*	0.0441*	0.0327*	0.0366*
Dec	0.0359*	0.0441*	0.0792*	0.0930*	0.0442*	0.0383*	0.0636*	0.0412*	0.0365*	0.0317*
Annual	0.0351*	0.0518*	0.0503*	0.0649*	0.0291*	0.0405*	0.0443*	0.0354*	0.0337*	0.0307*
**T**_**max**_										
Jan	0.0311*	0.0298*	0.0421*	−0.0013	0.0278*	0.0323	0.0377*	0.0131	0.0331*	0.0223
Feb	0.0277	0.0223*	0.0666	0.0282	0.0509*	0.0482*	0.0523*	0.0301*	0.0515*	0.0543*
Mar	0.0304*	0.0255*	0.0249*	0.0369*	0.0244	0.0123	0.0352*	0.0162	0.0131	0.0143
Apr	0.0021	0.0023	0.0180	0.0261*	0.0029	0.0275	0.0121*	0.0248	0.0141	0.0024
May	0.0097	0.0189*	0.0271	0.0466*	0.0092	0.0226	0.0236*	0.0252	0.0214*	0.0163
Jun	0.0203*	0.0148*	0.0333	0.0042	0.0229*	0.0223*	0.0397*	0.0136	0.0260*	0.0302*
Jul	0.0151*	0.0081*	0.0246	0.0270*	0.0131*	0.0197*	0.0275*	0.0137*	0.0184*	0.0101*
Aug	0.0203*	0.0153*	0.0293*	0.0171*	0.0193*	0.0154*	0.0353*	0.0150	0.0193*	0.0134
Sep	0.0174*	0.0132*	0.0379*	0.0229*	0.0183*	0.0203*	0.0270*	0.0131*	0.0258*	0.0226*
Oct	0.0179	0.0254*	0.0353*	0.0164	0.0153	0.0266*	0.0282*	0.0235*	0.0254	0.0205*
Nov	0.0389*	0.0426*	0.0477*	0.0546*	0.0274*	0.0393*	0.0380*	0.0338	0.0378*	0.0277*
Dec	0.0334*	0.0345*	0.0263*	0.0533*	0.0320*	0.0184	0.0312*	0.0013	0.0145*	0.0159*
Annual	0.0223*	0.0345*	0.0241*	0.0250*	0.0207*	0.0235*	0.0344*	0.0190*	0.0245*	0.0199*

Stars represent statistically significant trends (p < 0.05).

Mean monthly T_max_ ranges from −4.3 °C in January for IDR to 30.8 °C in June for TRB (Table 1). TRB shows the highest T_max_ during March – October among all basins, followed by NQMB during May - September. IDR and CTB display the lowest T_max_ during October – April, but in July and August T_max_ for IDR is higher than that for BPR and SWR. T_max_ is positive for all basins during March – November. YTR, YLR, MKR and BPR correspond to positive T_max_ throughout the year, indicating damped monthly T_max_ variations relative to the other basins.

By comparing monthly T_min_ and T_max_ for the same basin the monthly temperature range can be obtained. The largest temperature range of 17.7 °C occurs for BPR in December and the smallest range of 9.9 °C is for SWR in July. It is also found that all basins except TRB show relatively small monthly temperature ranges in the warm season (June - September) but large ranges in the cold season, especially in January and December. TRB, on the other hand, displays a large (small) range in August - October (December – February), indicating that TRB is in a climate zone different from the rest of the basins.

In terms of annual T_min_ and T_max_, not surprisingly, IDR and CTB are the coldest among all basins on the TPS. Conversely, YTR and TRB are the warmest on the TPS. The annual temperature range as represented by the mean annual T_max_ and T_min_ difference spans from 11.7 °C for YTR to 15.5 °C for IDR.

The trends of monthly T_min_ for the ten basins, ranging from 0.0165 °Cy^−1^ for MKR in June to 0.0939 °Cy^−1^ for IDR in November, are all positive and statistically significant (Table 2). The trends of monthly T_max_ are all positive except for a single negative trend of −0.0013 °Cy^−1^ for IDR in January; however, only about 1/3 of the positive trends are statistically significant (Table 2). With just a few exceptions, the trends of monthly T_min_ are consistently larger than those of T_max_ across the months and across the basins. Large increasing trends in monthly T_min_ are normally found in the cold season of November – February while large increasing trends in monthly T_max_ are noted mostly in February and November. The basins located in the northern TPS (CQB, NQMB, YLR, YTR and TRB) experience the largest increases in T_min_ in February, whereas the basins located in the southern TPS including CTB, MKR, SWR and BPR have the largest increases in T_min_ in December or January. For IDR, the positive trends in T_min_ are much larger during May – December than January – April, quite different from the seasonal evolution of the other basins. For T_max_, the largest increases occur in February for CTB, MKR, NQMB, SWR, YLR and YTR, and in November for BPR, CQB, IDR, SWR and TRB.

The trends of annual T_min_ and T_max_ are all positive and statistically significant for all basins (Table 2). The largest T_min_ (T_max_) trend, 0.0649 °Cy^−1^ (0.0345 °Cy^−1^), is found for IDR (CQB), the highest (coldest) basin; the smallest T_min_ (T_max_) trend, 0.0291 °Cy^−1^ (0.0190 °Cy^−1^), is found for MKR (TRB), located in the humid southeast (dry northwest). For the same basin, the increase rate for T_min_ is always larger than that for T_max_, demonstrating that there is more pronounced warming in T_min_ than in T_max_.

The annual T_min_ and T_max_ trends for TRB, 0.0354 °Cy^−1^ and 0.0190 °Cy^−1^, respectively, are very close to the trends of 0.0400 °Cy^−1^ and 0.0200 °Cy^−1^ reported by Lyu *et al*.^68^. Mamtimin *et al*.^69^ documented a warming trend of 0.1300 °C decade^−1^ (or 0.0130 °Cy^−1^) for TRB. The trends of annual T_min_ and T_max_ for YLR noted in this work are 0.0337 °Cy^−1^ and 0.0245 °Cy^−1^, respectively. Meng *et al*.^54^ found a warming rate of 0.3500 °C decade^−1^ or 0.0350 °Cy^−1^ and Jiang *et al*.^56^ obtained a warming rate of 0.0600 °Cy^−1^ for nearly the same basin. Positive trends for BPR were also reported in the literature, e.g., 0.0770–0.1540 °Cy^−1^ in 1988–2004^70^, and 0.0500 °Cy^−1^ in 1977–2006^71^. Clearly, warming trends prevail over the river basins of the TPS although it is difficult to compare the warming rates of T_min_ and T_max_ with the warming rates of mean temperature as normally reported by most other studies. Also, different studies use different study periods, different number of stations and methodology.

## Discussions

By focusing on monthly and annual temperature extremes on the TPS, we are able to reveal the spatiotemporal distributions of the extremes in great detail. The monthly temperature ranges (i.e., the differences between the maximum and the minimum) are smaller in June – September than in October – May for all basins except for the Tarim River basin where the opposite is largely true. The Tarim River basin also tends to experience the greatest temperature extremes among the ten basins, indicating a different climate condition for the Tarim River basin when compared to the other basins on the TPS.

This study demonstrates that on the TPS climate warming is happening and the trends of temperature extremes are increasing in recent decades, consistent with previous studies on the TP^44,49,72,73^, as well as in other regions^74–79^. On the TPS, both monthly and annual T_min_ and T_max_ display mostly increasing trends and the increasing trends are larger in magnitude than the decreasing trends that occur only sporadically, similar to what Keggenhoff *et al*.^25^ and Alexander *et al*.^31^ have reported. In agreement with what Rosmann *et al*.^80^ and Herath *et al*.^81^ have found, T_min_ especially extreme T_min_ increases more than T_max_ on the TPS, and thus T_min_ contributes more than T_max_ to climate warming in the region.

Accelerated warming, especially in winter and early spring, has been reported previously, e.g., Crabbe *et al*.^38^, Cuo *et al*.^42^, Cuo *et al*.^82^, Hu *et al*.^83^, Sun and Qin^84^ and Zhang *et al*.^85^, and is believed to be primarily due to some additional warming on top of the global warming. The additional warming may be partly related to the albedo effects caused by reduced snow and ice cover. On the TPS, February is the month of transition from deep winter to the beginning of spring and hence temperature is not as cold (or warm) as deep winter (or spring months). Due to the global warming, snow and ice that have been accumulated during the winter could melt faster in February compared to deep winter months. Hence the reduced albedo could enhance the warming of the air above the surface by absorbing more solar radiation and subsequently emitting more longwave radiation upward.

There are two possible reasons that may explain why T_min_ increasing rates are higher than T_max_ increasing rates on the TPS. First, the cloud amount has changed on the TPS in recent decades^86^, and changes in the cloud amount impact the energy balance and hence temperature^87^. Duan and Wu^85^ showed that in 1961–2003 a significant increase of the low-level cloud amount over the central and eastern TP during the nighttime leads to the enhanced atmospheric counter radiation and the weakened effective terrestrial radiation, which in turn gives rise to strong nocturnal surface warming (i.e., T_min_ warming). Duan and Wu^88^ further showed that decreases in both the total and low-level cloud amounts during the daytime result in more absorbing of direct solar radiation at the surface and the associated surface warming (i.e., T_max_ warming). However, the increase in nocturnal low-level cloud amount is greater than the decrease in daytime low and total cloud amounts, which lead to more increases in T_min_ than in T_max_^88^. Second, the mixture of light-scattering and light-absorbing aerosols produced from biomass burning, fossil fuel consumption and dust storms may also be a factor for relatively lower T_max_ increasing rate on the TPS, due to the scattering and absorption of daytime solar radiation by the aerosols^89–91^.

Although warming trends prevail over the TPS, cooling trends that occur sporadically are also noted. For example, April has the highest number of stations that display decreasing trends in monthly mean (35 stations out of the total 112 stations), extreme (29 stations), and the lowest (32 stations) maximum temperatures, which has not been reported by previous studies. The April anomaly (i.e., the temperature decreasing trends) concentrated in the southeastern TP may be a local phenomenon. Precipitation has increased in the southeastern TP during the same period. But whether or not precipitation change is responsible for the temperature decreasing trends there is still an open question and warrants further investigation in a future study. Also, besides Kuche in Xijiang, a few stations in the southern Tibet Autonomous Region and central Sichuan show negative trends in annual T_min_. Negative trends in annual T_max_ are concentrated in the northern Xinjiang and near the borders of the Tibet Autonomous Region, Yunnan and Sichuan. Station Yanyuan in Sichuan is clearly an outlier because statistically significant negative trends are frequently observed at this station for the monthly and annual T_max_ variables. The mechanisms behind the persistent negative trends in the monthly and annual temperature extremes certainly warrant further investigation in future study.

Liu and Chen^44^, Liu *et al*.^72^, and Yao *et al*.^91^, stated that the temperature warming rates on the TP increase with elevation. To examine the relationship between the warming rates of temperature extremes and elevation, we used 14, 27, 25, 38 and 8 stations in elevation ranges above 4000, 3000–4000, 2000–3000, 1000–2000 and less than 1000 m, respectively (the station locations and elevations are listed in the Supplementary Table S1). The scatter plots of elevations vs. trends (not shown) indicate positive correlations to some extent for most cases but the correlations are not statistically significant and the coefficients of determination (R^2^) are much less than 0.1 for all annual cases and most monthly cases. The exceptions are: R^2^ = 0.1109 (0.3577) for monthly T_max_ in January (December), R^2^ = 0.1509 (0.1444, 0.3162) for monthly extreme T_max_ in January (November, December), R^2^ = 0.2289 (0.1979 and negative correlation) for monthly lowest T_max_ in September (October), and R^2^ = 0.1593 for monthly highest T_min_ in January, and all of the associated correlations are statistically significant at *p* < 0.05 except for monthly extreme T_max_ in November. Hence, it appears that the annual warming rates of temperature extremes and elevation do not show statistically robust positive correlations over the TPS, in agreement with Cuo *et al*.^49^ and You *et al*.^92^, but contrary to Liu and Chen^44^, Liu *et al*.^72^ and Yao *et al*.^93^ who examined the warming rates of mean temperature rather than temperature extremes. On the other hand, in winter months, the warming rates of some T_max_ variables and the highest T_min_ do appear to correlate with elevation in a statistically significant way.

The possible reasons for the disagreement in the elevation dependence of the warming rates may be related to the differences in data and the analysis approaches. First, in Liu and Chen^44^, Liu *et al*.^72^ and Yao *et al*.^93^, temperatures were averaged for elevation zones based on station elevations and this could smooth out the temperature spatial variability within a cluster of individual stations, which has the potential issue of comparing elevation zones with different number of stations averaged as well as insufficient representativeness of temperature warming rates in elevation zones with fewer stations. Second, the aforementioned three studies drew conclusions without testing the statistical significance, which could cause discrepancies when the correlations are not statistically significant.

Given the fact that glaciers have much higher warming rates than low elevation as shown by Yao *et al*.^93^, Tian *et al*.^94^ and Kang *et al*.^95^, it is possible that high warming rates may have only occurred in glaciers and snow capped high mountains whereas in the other high elevation with no snow or glaciers the warming rates may still be comparable to those of low elevation.

What differs this study from the previous ones and also our new findings can be summarized as follows. First, previous studies mostly focused on the annual and seasonal time series of temperature minima and maxima and other climate elements on the Tibetan Plateau only, not on monthly time series, let alone the spatial patterns of extreme temperatures over the Tibetan Plateau and its surroundings (TPS). By examining the extreme temperature variables at monthly time scale and over a larger area, this study is able to reveal detailed and unique spatial patterns of monthly conditions and changes of extreme temperatures over an extended period of time. Second, most previous studies show that winter temperature increase rate is the highest among all seasons. This study finds that the highest warming rates in extreme temperatures tend to occur in February specifically. Third, this study is the first to document that about one third of the stations show negative trends in extreme T_max_ variables in April. Fourth, this study examines and compares the averaged monthly and annual temperature conditions among the ten river basins which has not been reported before. Fifth, this study provides new and detailed insights into the relationship between extreme temperature variables and elevation. Clearly, this study presents a comprehensive and detailed analysis of the regional, basin and monthly differences in climate conditions and changes, which is important not only for climate model validation but also for the application and modeling of water resources and ecological systems.

## Conclusions

Annual and monthly temperature extremes, cold and warm event indices and their changes during 1963–2015 on the TPS are investigated. The coldest area is the central TPS while the hottest area is the southeastern TPS in the annual sense and the northwestern TPS in the summer months. The northwest (southeast) displays the highest (lowest) monthly extreme temperature ranges. About 87% and 71% stations show positive trends for monthly T_min_ and T_max_, respectively. February features the most positive trends for both T_min_ and T_max_ among all the months. For the monthly T_min_ variables, statistically significant negative trends are found mainly in May and July in the northwestern TPS and near the borders of T, S and Y. For the monthly T_max_ variables, negative trends are noted primarily in S especially in April. About 95–96% of stations show positive trends for annual T_min_ and T_max_, and 94–96% of the positive trends are statistically significant. In general, warm events frequent at Yibin, Leshan and Dujiangyan in S while cold events often occur at Wudaoliang (52908), Qingshuihe (56034) and Tuotuohe (56004) in Q and Tuergate (51701) in X.

Over the ten river basins, the trends of monthly T_min_ are all positive and statistically significant while the trends of monthly T_max_ are all positive except for one negative trend for IDR in January, but only around 1/3 of the positive trends are statistically significant. The trends of annual T_min_ and T_max_, ranging from the highest 0.0649 °Cy^−1^ (T_min_) for IDR and 0.0345 °Cy^−1^ (T_max_) for CQB to the lowest 0.0291 °Cy^−1^ (T_min_) for MKR and 0.0190 °Cy^−1^ (T_max_) for TRB, are all positive and statistically significant for all basins. The warming rates of annual temperature extremes do not appear to depend strongly on elevation on the TPS, whereas the warming rates of some T_max_ variables and the highest T_min_ correlate robustly with elevation in winter months.

## Electronic supplementary material

Supplementary Materials
