# Supplementary material for: Monthly and annual temperature extremes and their changes on the Tibetan Plateau and its surroundings during 1963–2015
Source: Sci Rep. 2018 Aug 8;8:11860. doi: 10.1038/s41598-018-30320-0 (PMC6082912; doi:10.1038/s41598-018-30320-0)
Supplement: Supplementary file 1 — Supplementary Materials [file 41598_2018_30320_MOESM1_ESM.pdf]

Supplementary Materials to

Monthly and annual temperature extremes and their changes on the Tibetan Plateau and its surroundings during 1963-2015

Jin Ding[1, 2], Lan Cuo[1, 2, 3*], Yongxin Zhang[4], and Fuxin Zhu[1, 2]

[1] *Key Laboratory of Tibetan Environment Changes and Land Surface Processes, Institute of Tibetan Plateau Research, Chinese Academy of Sciences, Beijing, China.*

[2] *University of Chinese Academy of Sciences, Beijing, China.*

[3] *Center for Excellence in Tibetan Plateau Earth Sciences, Beijing, China.*

[4] *Research Applications Laboratory and Climate and Global Dynamics Laboratory, National Center for Atmospheric Research, Boulder, Colorado, USA.*

Table S1. The weather stations used in the study.

ID	Longitude	Latitude	Elevation (m)	Station name	Province	Basin it is located
55493	91.10000	30.48333	4200.0	Dangxiong	T	BPR
55578	88.88333	29.25000	3836.0	Rikaze	T	BPR
55598	91.76667	29.25000	3551.7	Zedang	T	BPR
55696	92.46667	28.41667	3860.0	Longzi	T	BPR
55773	89.08333	27.73333	4300.0	Pali	T	BPR
56202	93.28333	30.66667	4488.8	Jiali	T	BPR
56227	95.76667	29.86667	2736.0	Bomi	T	BPR
56312	94.33333	29.66667	2991.8	Linzhi	T	BPR
51886	90.85000	38.25000	2944.8	Mangai	Q	CQB
52602	93.33333	38.75000	2770.0	Lenghu	Q	CQB

52713	95.36667	37.85000	3173.2	Dachaidan	Q	CQB
52754	100.13333	37.33333	3345.0	Gangcha	Q	CQB
52825	96.41667	36.43333	2790.4	Nuomuhong	Q	CQB
52836	98.10000	36.30000	3191.1	Dulan	Q	CQB
55279	90.01667	31.38333	4700.0	Bange	T	CTB
55472	88.63333	30.95000	4672.0	Shenzha	T	CTB
55228	80.08333	32.50000	4278.6	Shiquanhe	T	IDR
56018	95.30000	32.90000	4066.4	Zaduo	Q	MKR
56125	96.48333	32.20000	3643.7	Nangqian	Q	MKR
56137	97.16667	31.15000	3306.0	Changdu	T	MKR
56548	99.28333	27.16667	2326.1	Weixi	Y	MKR
56751	100.18333	25.70000	1990.5	Dali	Y	MKR
52313	94.66667	41.53333	1573.8	Hongliuhe	X	NQMB
52546	99.83333	39.36667	1332.2	Gaotai	G	NQMB
52633	98.41667	38.80000	3367.0	TuoLe	Q	NQMB
52645	99.58333	38.41667	3537.0	Yeniugou	Q	NQMB
52652	100.43333	38.93333	1461.1	Zhangye	G	NQMB
52657	100.25000	38.18333	2787.4	Qilian	Q	NQMB
52661	101.08333	38.80000	1764.6	Shandan	G	NQMB
52674	101.96667	38.23333	1976.1	Yongchang	G	NQMB
52679	102.66667	37.91667	1530.9	Wuwei	G	NQMB

52681	103.08333	38.63333	1367.5	Minqin	G	NQMB
55299	92.06667	31.48333	4507.0	Naqu	T	SWR
56106	93.78333	31.88333	4022.8	Suoxian	T	SWR
56444	98.91667	28.48333	3319.0	Deqin	Y	SWR
56748	99.18333	25.11667	1652.2	Baoshan	Y	SWR
51628	80.23333	41.16667	1103.8	Akesu	X	TRB
51633	81.90000	41.78333	1229.2	Baicheng	X	TRB
51642	84.25000	41.78333	976.1	Luntai	X	TRB
51644	82.96667	41.71667	1081.9	Kuche	X	TRB
51656	86.13333	41.75000	931.5	Luerle	X	TRB
51701	75.40000	40.51667	3504.4	Tuergate	X	TRB
51705	75.25000	39.71667	2175.7	Wuqia	X	TRB
51711	78.45000	40.93333	1984.9	Aheqi	X	TRB
51720	79.05000	40.50000	1161.8	Keping	X	TRB
51730	81.26667	40.55000	1012.2	Alaer	X	TRB
51765	87.70000	40.63333	846.0	Tieganlike	X	TRB
51777	88.16667	39.03333	887.7	Ruoqiang	X	TRB
51804	75.23333	37.76667	3090.1	Tashenkuergan	X	TRB
51811	77.26667	38.43333	1231.2	Shache	X	TRB
51818	78.28333	37.61667	1375.4	Pishan	X	TRB
51828	79.93333	37.13333	1375.0	Hetian	X	TRB

51839	82.71667	37.06667	1409.5	Minfeng	X	TRB
51855	85.55000	38.15000	1247.2	Qiemo	X	TRB
51931	81.65000	36.85000	1422.0	Yutian	X	TRB
52765	101.61667	37.38333	2938.0	Mengyuan	Q	YLR
52787	102.86667	37.20000	3045.1	Wuqiaoling	G	YLR
52797	104.05000	37.18333	1630.5	Jingtai	G	YLR
52856	100.61667	36.26667	2835.0	Qiabuqia	Q	YLR
52868	101.43333	36.03333	2237.1	Guide	Q	YLR
52876	102.85000	36.31667	1813.9	Minhe	Q	YLR
52895	104.68333	36.56667	1398.2	Jinyuan	G	YLR
52943	99.98333	35.58333	3323.2	Xinghai	Q	YLR
52983	104.15000	35.86667	1874.4	Yuzhong	G	YLR
52984	103.18333	35.58333	1917.0	Lingxia	G	YLR
52986	103.85000	35.35000	1886.6	Lintao	G	YLR
52996	105.00000	35.38333	2450.6	Huajialing	G	YLR
56046	99.65000	33.75000	3967.5	Dari	Q	YLR
56067	101.48333	33.43333	3628.5	Jiuzhi	Q	YLR
56079	102.96667	33.58333	3439.6	Ruoergai	S	YLR
56080	102.90000	35.00000	2910.0	Hezuo	G	YLR
56093	104.01667	34.43333	2315.0	Minxian	G	YLR
52908	93.08333	35.21667	4612.2	Wudaoliang	Q	YTR

56004	92.43333	34.21667	4533.1	Tuotuohe	Q	YTR
56021	95.78333	34.13333	4175.0	Qumalai	Q	YTR
56029	97.01667	33.01667	3681.2	Yushu	Q	YTR
56034	97.13333	33.80000	4415.4	Qingshuihe	Q	YTR
56038	98.10000	32.98333	4290.0	Shiqu	S	YTR
56144	98.58333	31.80000	3328.0	Dege	S	YTR
56146	100.00000	31.61667	3393.5	Ganzi	S	YTR
56152	100.33333	32.28333	3893.9	Seda	S	YTR
56167	101.11667	30.98333	2957.2	Daofu	S	YTR
56172	102.23333	31.90000	2664.4	Maerkang	S	YTR
56173	102.55000	32.80000	3491.6	Hongyuan	S	YTR
56178	102.35000	31.00000	2369.2	Xiaojin	S	YTR
56182	103.56667	32.65000	2850.7	Songpan	S	YTR
56188	103.66667	31.00000	698.5	Dujiangyan	S	YTR
56193	104.51667	32.41667	893.2	Pingwu	S	YTR
56251	100.31667	30.93333	3091.0	Xinlong	S	YTR
56357	100.30000	29.05000	3727.7	Daocheng	S	YTR
56374	101.96667	30.05000	2615.7	Kangding	S	YTR
56385	103.33333	29.51667	3047.4	Emeishan	S	YTR
56386	103.75000	29.56667	424.2	Leshan	S	YTR
56459	101.26667	27.93333	2426.5	Muli	S	YTR

56462	101.50000	29.00000	2925.0	Jiulong	S	YTR
56475	102.51667	28.65000	1659.5	Yuexi	S	YTR
56479	102.85000	28.00000	2132.4	Zhaojue	S	YTR
56485	103.58333	28.26667	1255.8	Leibo	S	YTR
56492	104.60000	28.80000	340.8	Yibin	S	YTR
56543	99.70000	27.83333	3276.7	Xianggelila	Y	YTR
56565	101.51667	27.43333	2545.0	Yanyuan	S	YTR
56571	102.26667	27.90000	1590.9	Xichang	S	YTR
56586	103.71667	27.35000	1949.5	Zhaotong	Y	YTR
56651	100.21667	26.86667	2380.9	Lijiang	Y	YTR
56671	102.25000	26.65000	1787.3	HuiLi	S	YTR
56684	103.28333	26.41667	2188.3	Huize	Y	YTR
56768	101.55000	25.03333	1824.1	Chuxiong	Y	YTR
56778	102.65000	25.00000	1888.1	Kunming	Y	YTR
56786	103.83333	25.58333	1898.7	Zhanyi	Y	YTR
52533	98.48333	39.76667	1477.2	Jiuquan	G	
56533	98.66667	27.75000	1583.3	Gongshan	Y	
56739	98.50000	25.01667	1695.9	Tengchong	Y	

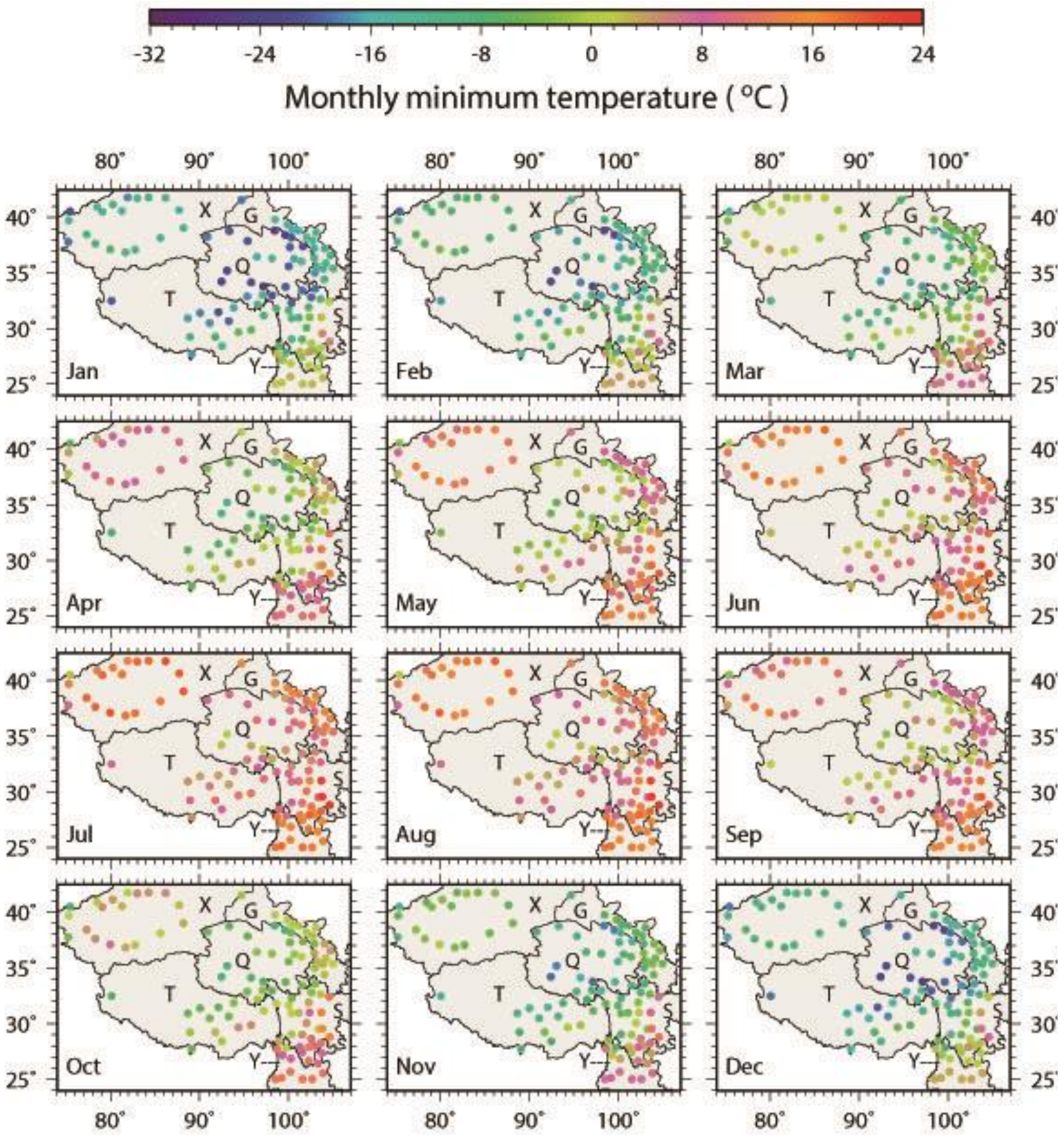

Figure S1. The spatial distributions of monthly minimum temperature.

Monthly T_{min} ranges from -26.0 to 23.6°C across all months and stations (Fig.S1). The lowest (highest) monthly T_{min} occurs in January (July and August). The spatial pattern of monthly T_{min} generally follows elevation all year round but especially so in November – March, with the coldest and warmest T_{min} located in the high elevation of the central TPS and low elevation of the southeastern TPS, respectively. Monthly T_{min} in Q and T, which together make up the major part of the TPS and where the high

mountains are also located, is about 10.0 - 20.0°C lower than the northwestern TPS (X) and about 10.0 - 30.0°C lower than the southeastern TPS (Y and the southern S). Monthly T_{min} below 7.0°C always occurs in the central TPS (i.e., T and Q) from January to December. Generally, the southeast is about 10.0 - 20.0°C warmer than the northwest (X) in October - March primarily because of the effect of latitudes. On the other hand, monthly T_{min} in the southeast and northwest exhibits little difference in April – September largely due to the fact that the southeast features lush green vegetation and a high amount of precipitation which consume more net radiation for latent heat flux than for sensible heat flux while the northwest is normally barren and dry and more energy is available for sensible heat flux.

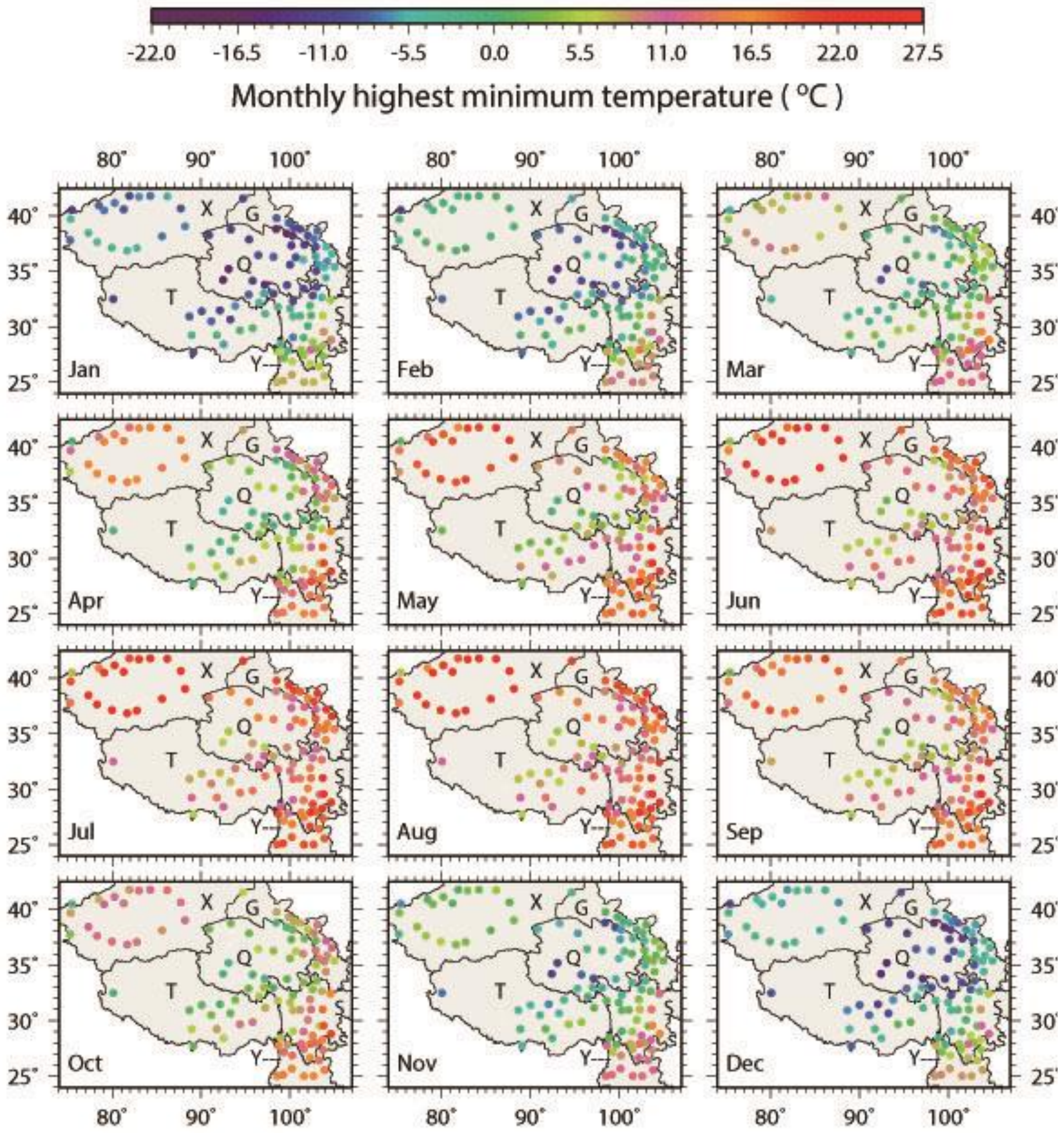

Figure S2. The spatial distributions of monthly highest minimum temperature.

Monthly highest T_{min} (Fig.S2) exhibits rather similar spatial and temporal distributions to monthly T_{min} and monthly extreme T_{min}, with the central TPS colder than the northwest and southeast, and January (July and August) being the coldest (warmest) month(s). Monthly highest T_{min} ranges from -17.0 to 27.2°C. The southeast, whose monthly highest T_{min} is ≥ 0.0°C in all twelve months, is the warmest on the TPS. The largest differences in monthly highest T_{min} are seen in the northwest and the central

TPS (in Q and T), reaching about 30.0°C between summer and winter for both regions. All three minimum temperatures examined above show consistent spatiotemporal variations that are influenced by elevation, latitudes, land cover, prevailing weather systems, and the seasonal march of the Sun.

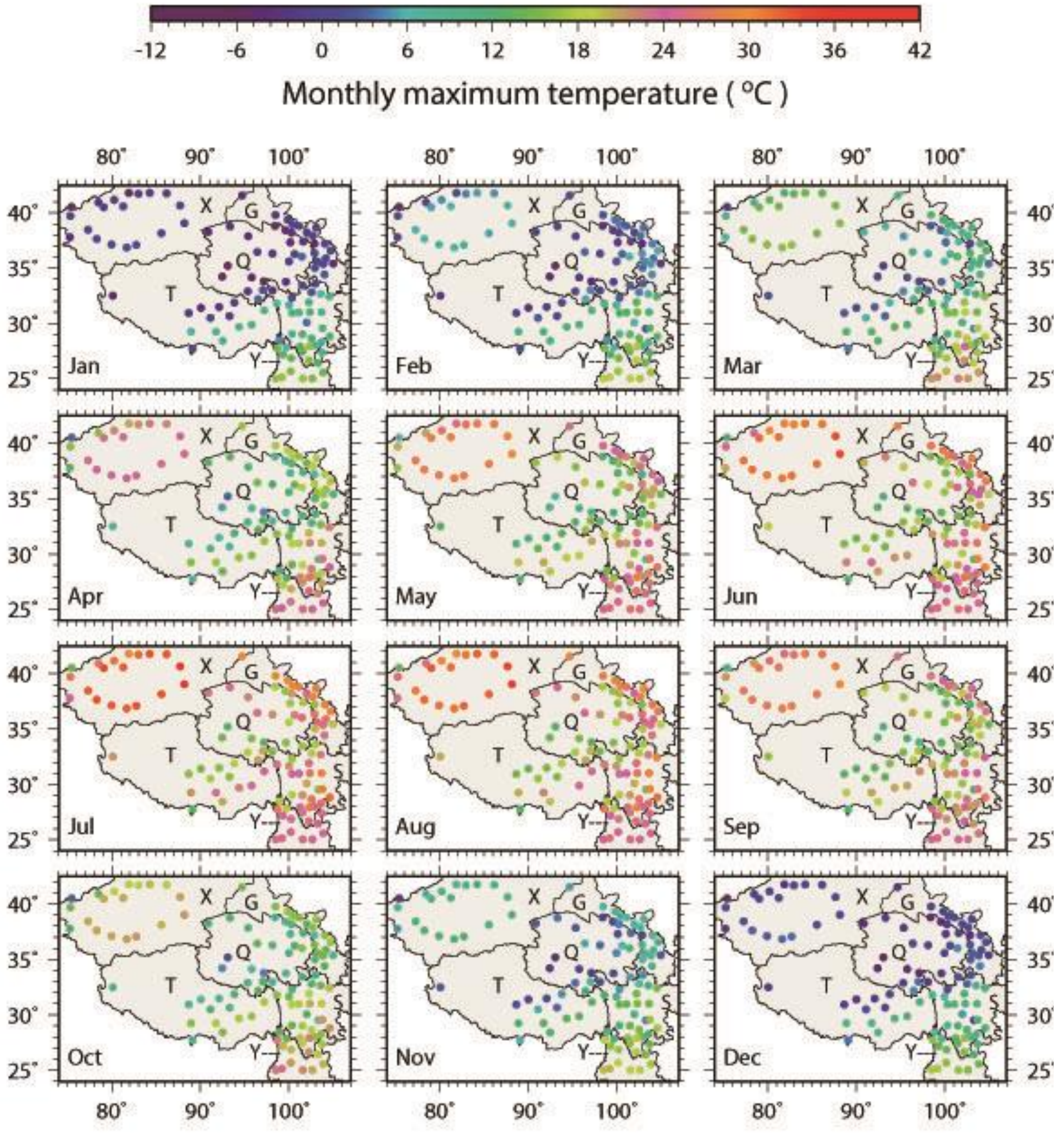

Figure S3. The spatial distributions of monthly maximum temperature.

Monthly T_{max} ranges from -8.4 to 36.0°C across the TPS, from 6.0 to 30.0°C in

the southeast and from -6.0 to 36.0°C in the northwest between January and July/August (Fig.S3). Similar to monthly T_{min}, the lowest monthly T_{max} occurs in the central TPS in January and December but contrary to monthly T_{min}, the highest monthly T_{max} is found in the northwest in July and August (instead of the southeast for monthly T_{min}). The differences in monthly T_{max} between the coldest and warmest areas on the TPS stay around 20.0°C which is about 10.0°C lower than those in monthly T_{min}.

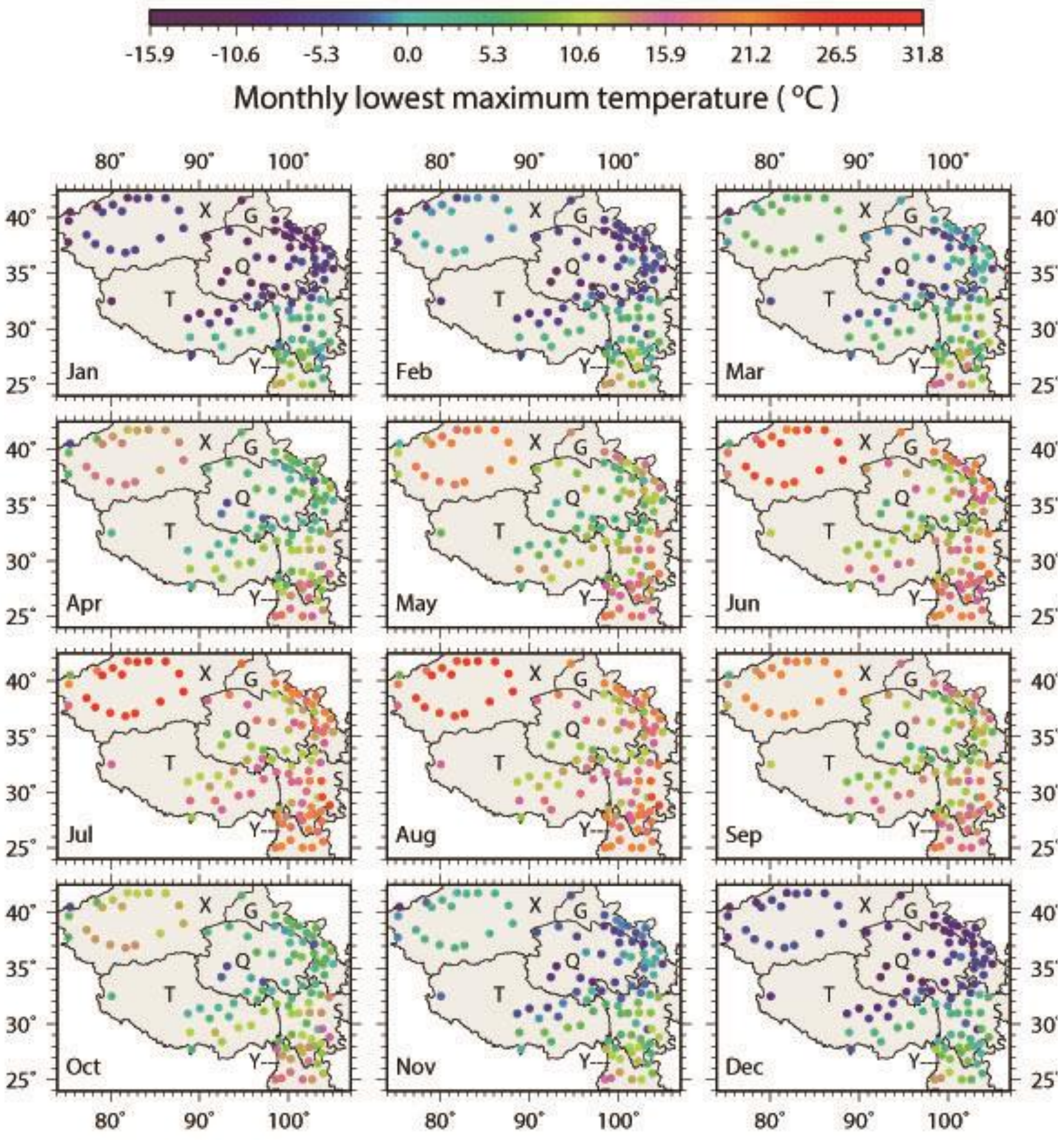

Figure S4. The spatial distributions of monthly lowest maximum temperature.

Monthly lowest T_{max} ranges from -16.0°C at Tuergate (51701) to 29.0°C at Ruoqiang (51777) in X, and generally the lowest values are found in January and December in the central TPS and the highest values in July and August in the northwest (Fig.S4). Monthly lowest T_{max} is always greater than 30.0°C during June - August in the northwest but lower than -20.0°C during October - April in the central TPS. Compared with Fig.3, it can be seen that in winter, the range of monthly T_{max} is around -18.0 - -6.0°C in the northwest and central TPS, and 10.0 - 20.0°C in the southeast. In

summer, the range becomes about 28.0 - 48.0°C in the northwest, 6.0 - 25.0°C in the central TPS, and 15.0 - 35.0°C in the southeast.

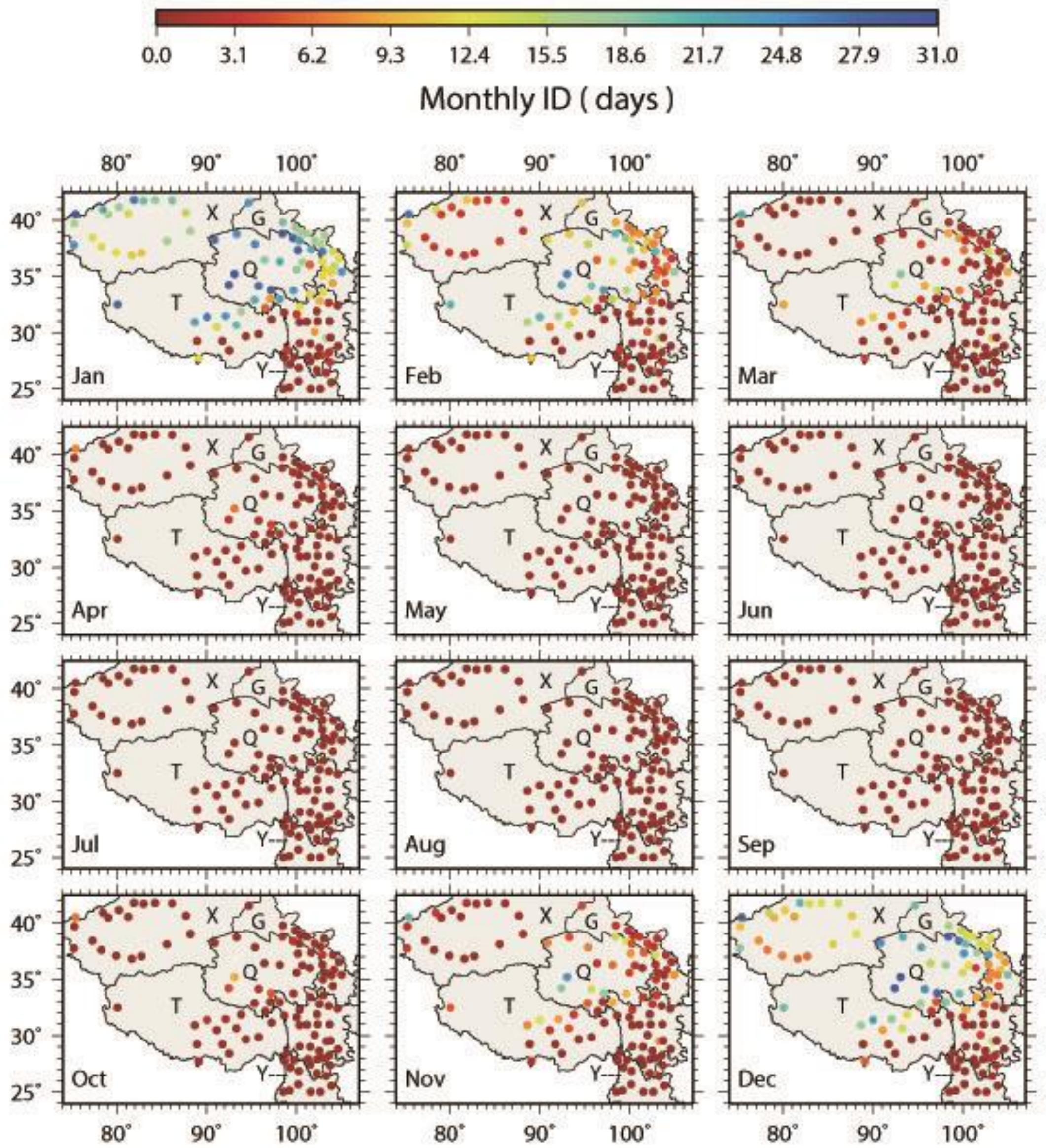

Figure S5. The spatial distributions of monthly icing days.

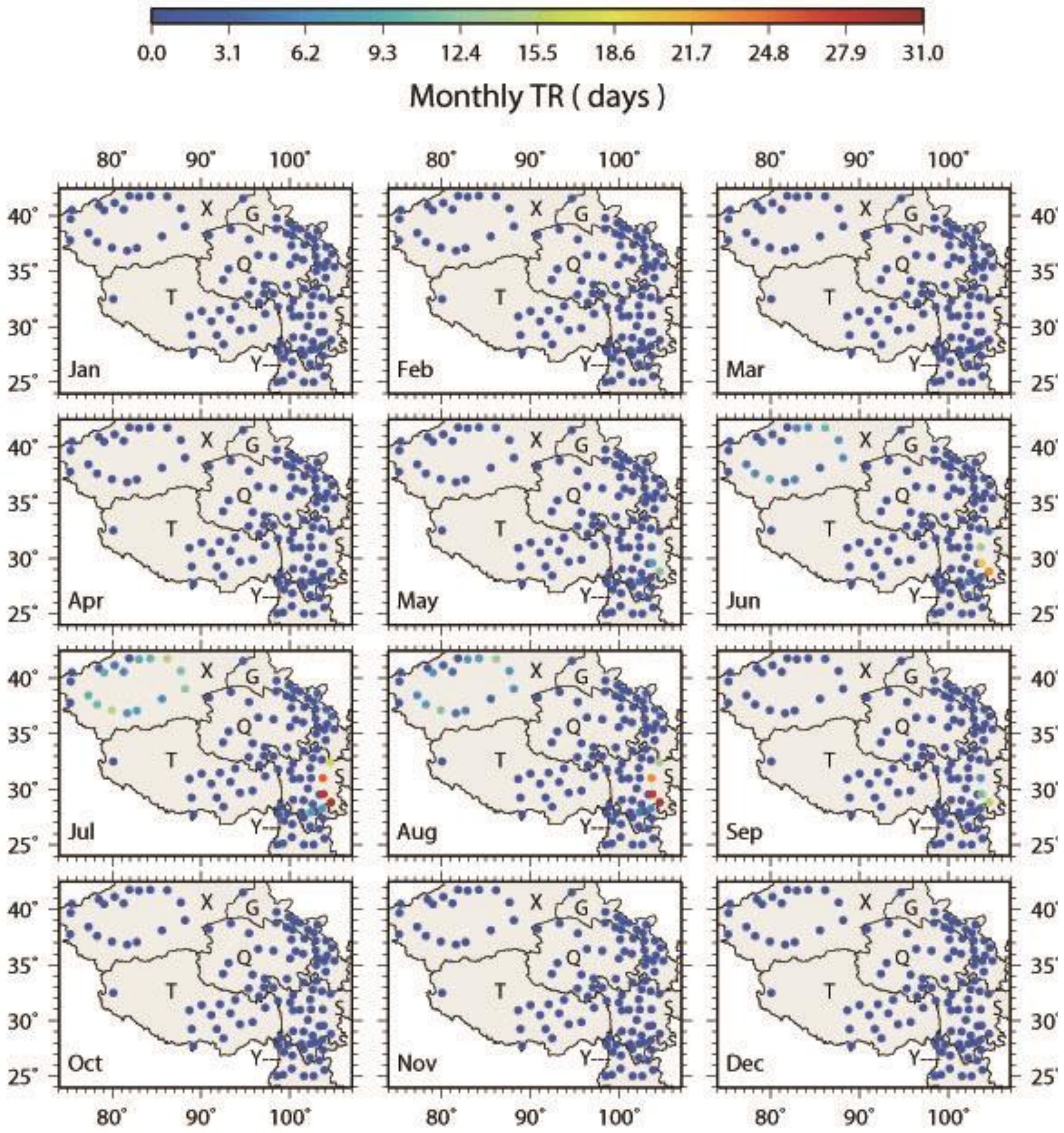

Figure S6. The spatial distributions of monthly tropical night days.

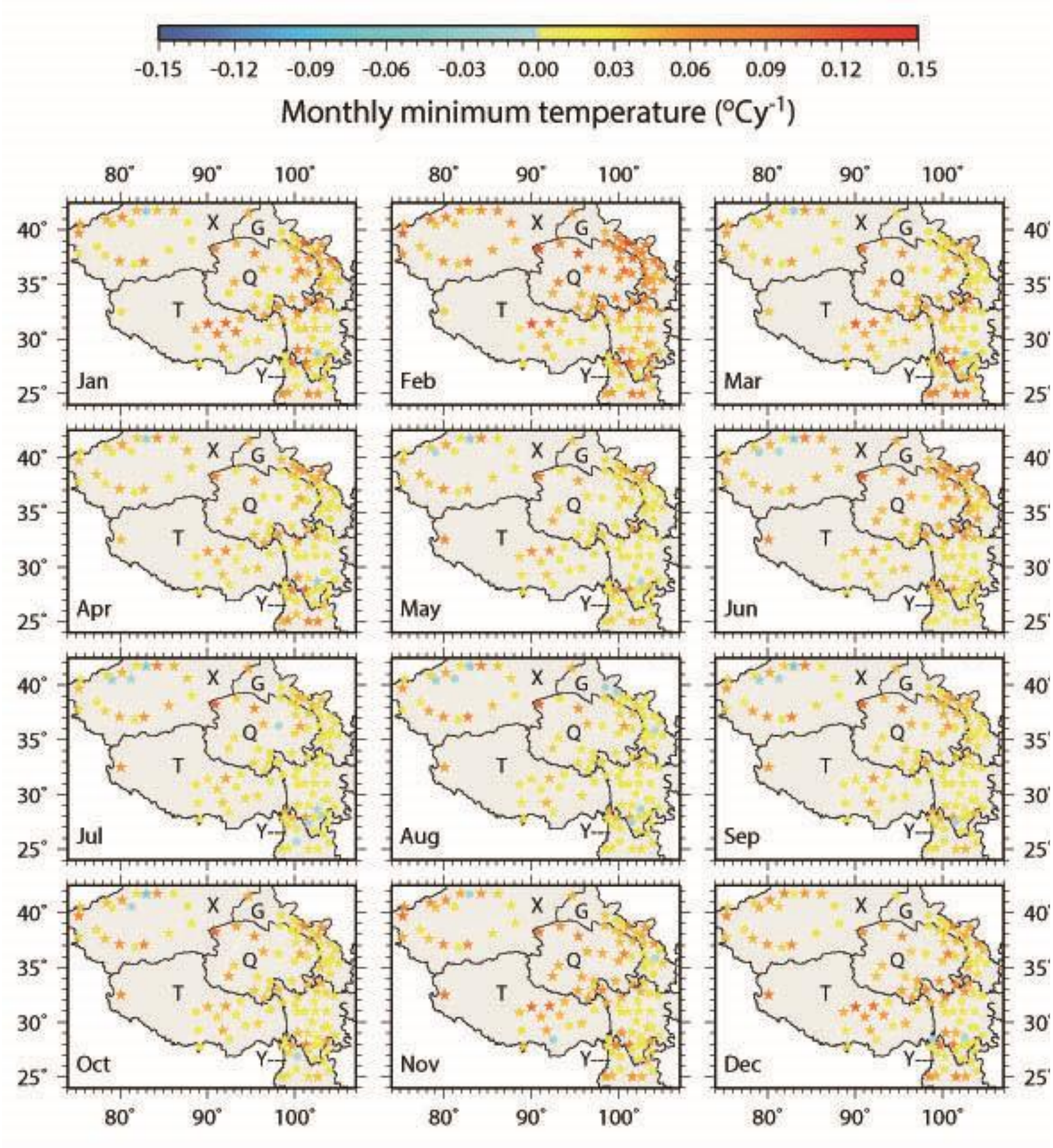

Figure S7. The spatial distributions of the trends of monthly minimum temperature during 1963-2015. Black circles represent zero trends. Stars represent statistically significant trends (p<0.05).

The trends of monthly T_{min} range from -0.0540 °Cy^{-1} at Kuche (51644 in X) in September to 0.1300 °Cy^{-1} at Shandan (52661 in G) in February (Fig.S7). The majority of the trends are positive throughout the year on the TPS, in line with global warming. Amongst the months, February features all positive and also the largest trends, with 94% of the stations showing statistically significant trends. In comparison, July and August

correspond to relatively smaller trends, and July features eight stations with negative trends among which five are statistically significant. Spatially, monthly T_{min} displays the highest and statistically significant positive trends over the northeast (G and part of Q), especially in February and June. The trends over the southeast are generally small throughout the year. There appears to be a tendency for the trends of monthly T_{min} to decrease gradually from the northwest to the southeast especially in the warm season. During the cold season (November - March), the central T and the southern Y display more statistically significant increasing trends of ~0.1000 °Cy^{-1} than the other areas.

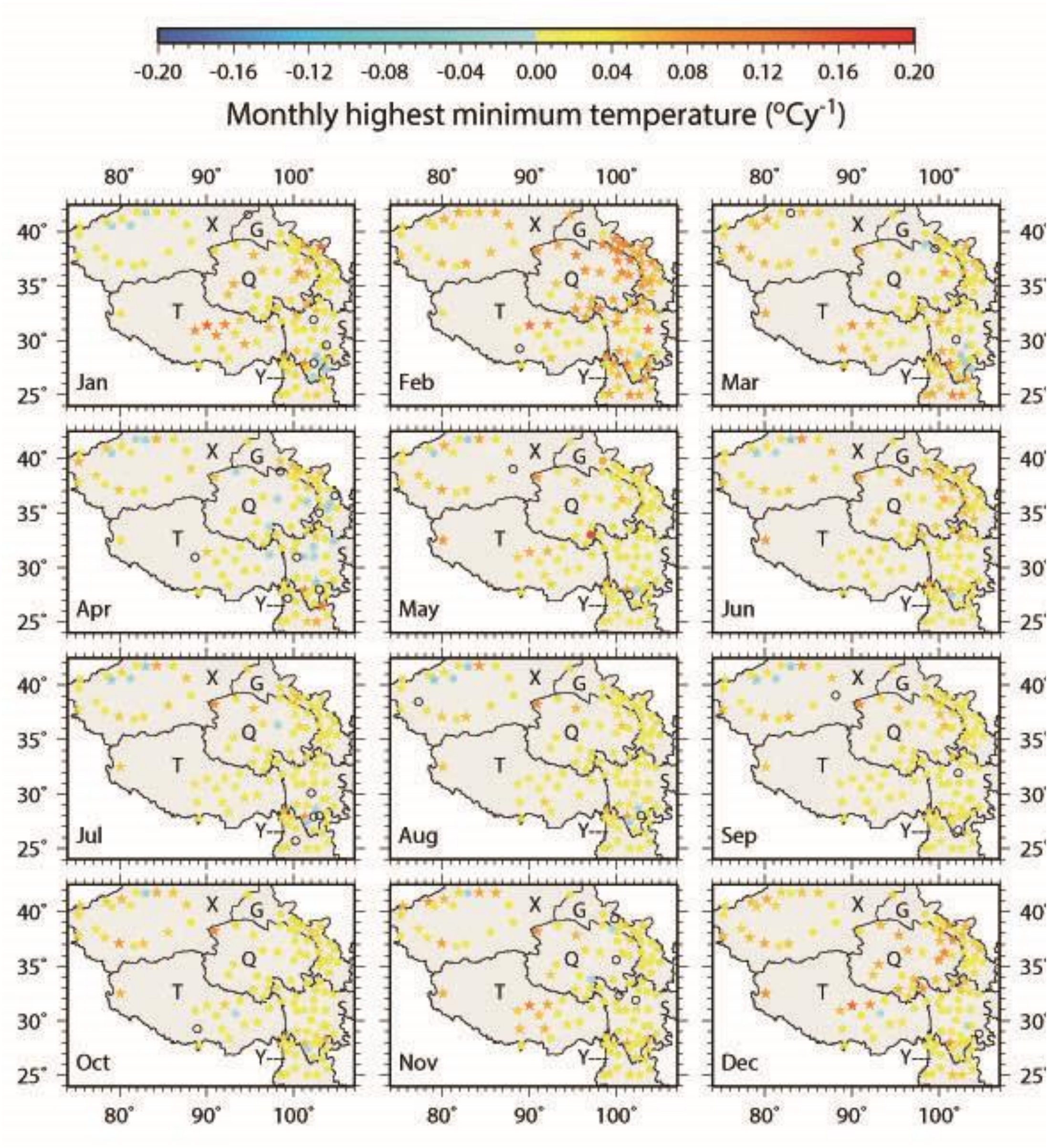

Figure S8. The spatial distributions of the trends of monthly highest minimum temperature during 1963-2015. Black circles represent zero trends. Stars represent statistically significant trends ($p<0.05$).

Similar to monthly T_{min} and monthly extreme T_{min}, monthly highest T_{min} also displays predominantly increasing trends across the TPS and throughout the year, with normally less than 10 stations showing decreasing trends during any month (Fig.S8). The trends of monthly highest T_{min} range from -0.0270 °Cy^{-1} at Kuche (51644 in X) in June to 0.1800 °Cy^{-1} at Yushu (56029 in Q) in May. Statistically significant decreasing

trends are noted mainly during April – September, e.g., -0.0111 °Cy^{-1} at Yuexi (56475) in April, -0.0270 °Cy^{-1} at Kuche (51644) in June, and -0.0250 °Cy^{-1} at Keping (51720) in August. Smaller trends are generally found in the eastern part of the TPS when compared to the northwest and central TPS except for February. During December - February, the central T corresponds to large and statistically significant increasing trends in the range of 0.1300 to 0.1800 °Cy^{-1}.

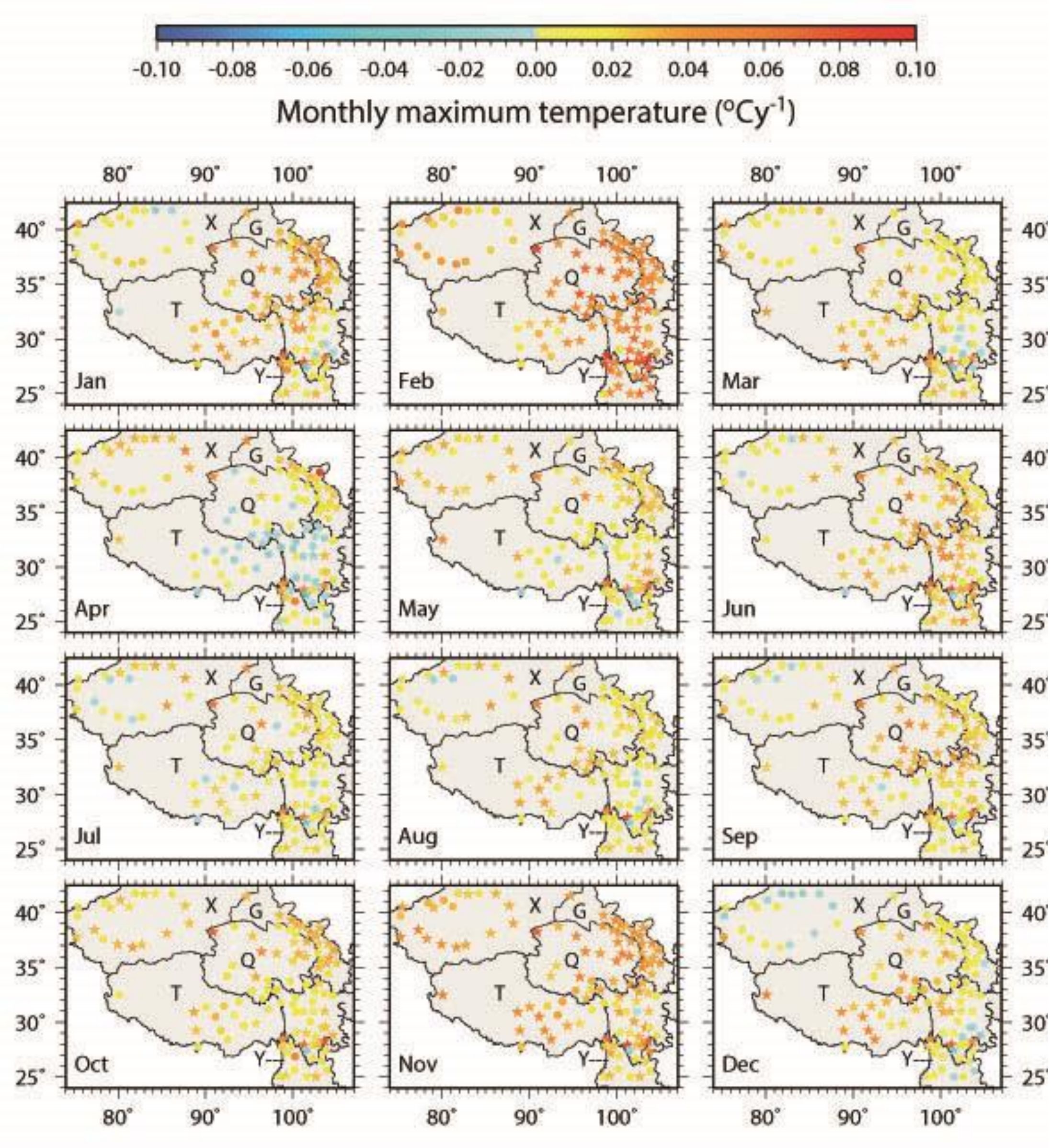

Figure S9. The spatial distributions of the trends of monthly maximum temperature during 1963-2015. Black circles represent zero trends. Stars represent statistically significant trends (p<0.05).

The trends of monthly T_{max} ranges from -0.0450 °Cy^{-1} at Yanyuan (56565 in S) to 0.1000 °Cy^{-1} at Muli (56459 in S), and the trends are positive at the majority of the stations throughout the year (Fig.S9). Most statistically significant positive trends are found in November, February and June at 75%, 69% and 69% of the stations, respectively. Similar to monthly T_{min}, monthly T_{max} shows increasing trends at all

stations in February. The decreasing trends of monthly T_{max}, located mainly along the borders of T, X, Q and S, are noted primarily in April and to a lesser extent in December, July and January. Among the 1/3 of the stations that correspond to the decreasing trends in April, only two stations in S, Xiaojin (56178, -0.0203 °Cy^{-1}) and Yanyuan (56565, -0.0443 °Cy^{-1}) show statistically significant trends. Unlike the monthly T_{min} variables that display some sort of spatial coherence by clustering large trends in the central T, large monthly T_{max} trends tend to spread out across the TPS in different months.

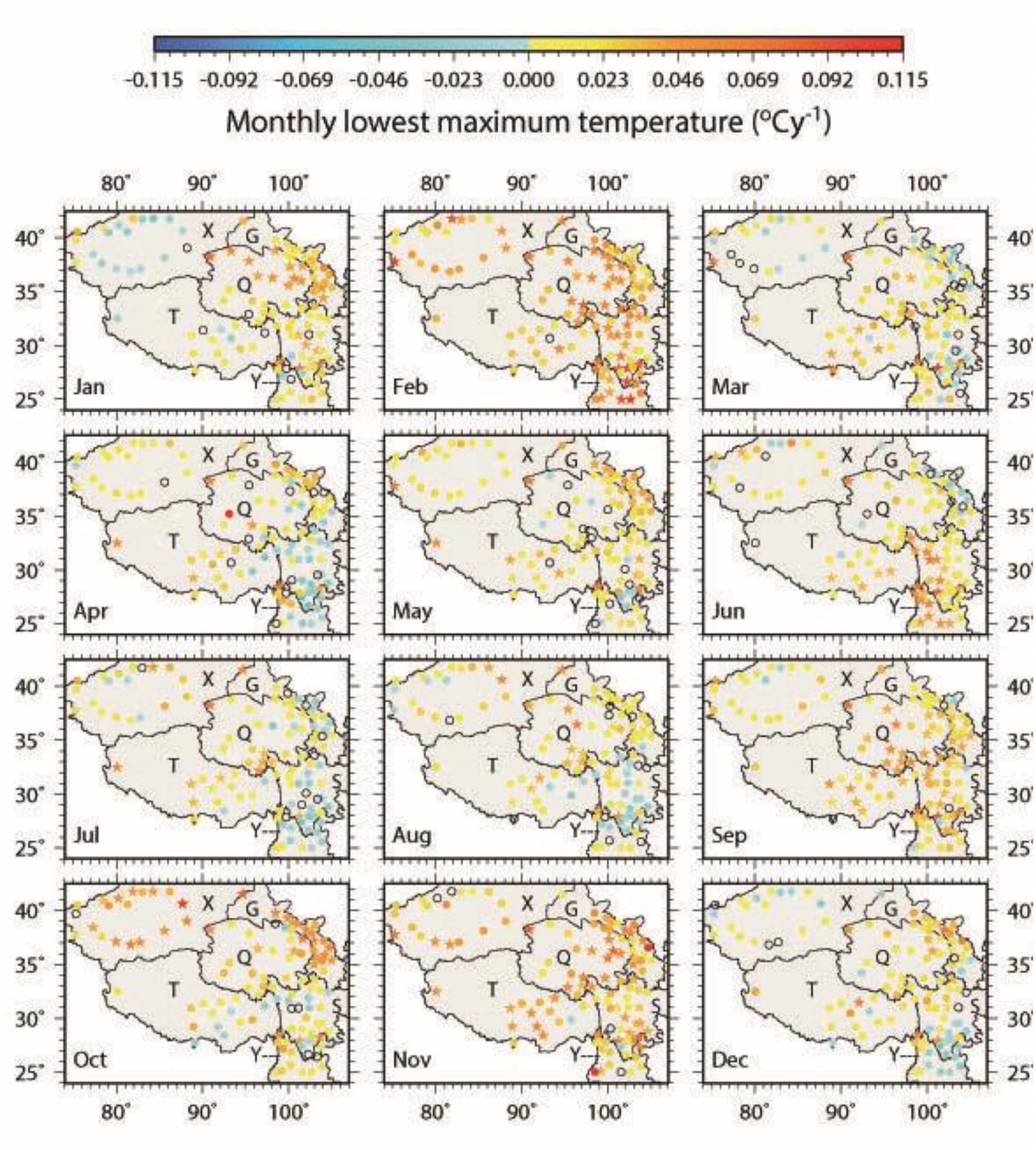

Figure S10. The spatial distributions of the trends of monthly lowest maximum temperature during 1963-2015. Black circles represent zero trends. Stars represent statistically significant trends (p<0.05).

The trends of monthly lowest T_{max} range from -0.0460 °Cy^{-1} at Yanyuan (56565 in S) to 0.1150 °Cy^{-1} at Kunming (56778 in Y) (Fig.S10). Similar to monthly T_{max} and monthly extreme T_{max}, monthly lowest T_{max} exhibits the largest and the most number of statistically significant increasing trends in February near the borders of S and Y as

well as in the central Y; while the decreasing trends are found in April, July and August in the eastern TPS and in December and January in the northwestern TPS. In February, 41% of the increasing trends are statistically significant and the number is 35% and 34% for September and November, respectively. 32%, 32% and 25% of all stations correspond to decreasing trends in April, July and August, respectively, and April features the largest decreasing trends in magnitude. All statistically significant negative trends occur in S and in the warm season, e.g., -0.0279 °Cy^{-1} in July at Leshan (56386), -0.0460 °Cy^{-1} in July and -0.0394 °Cy^{-1} in August at Yanyuan (56565), -0.0294 °Cy^{-1} in July at Xichang (56571), and -0.0350 °Cy^{-1} in August at Yuexi (56475). Among all monthly temperature variables, the lowest T_{max} shows the smallest and the fewest significant trends.

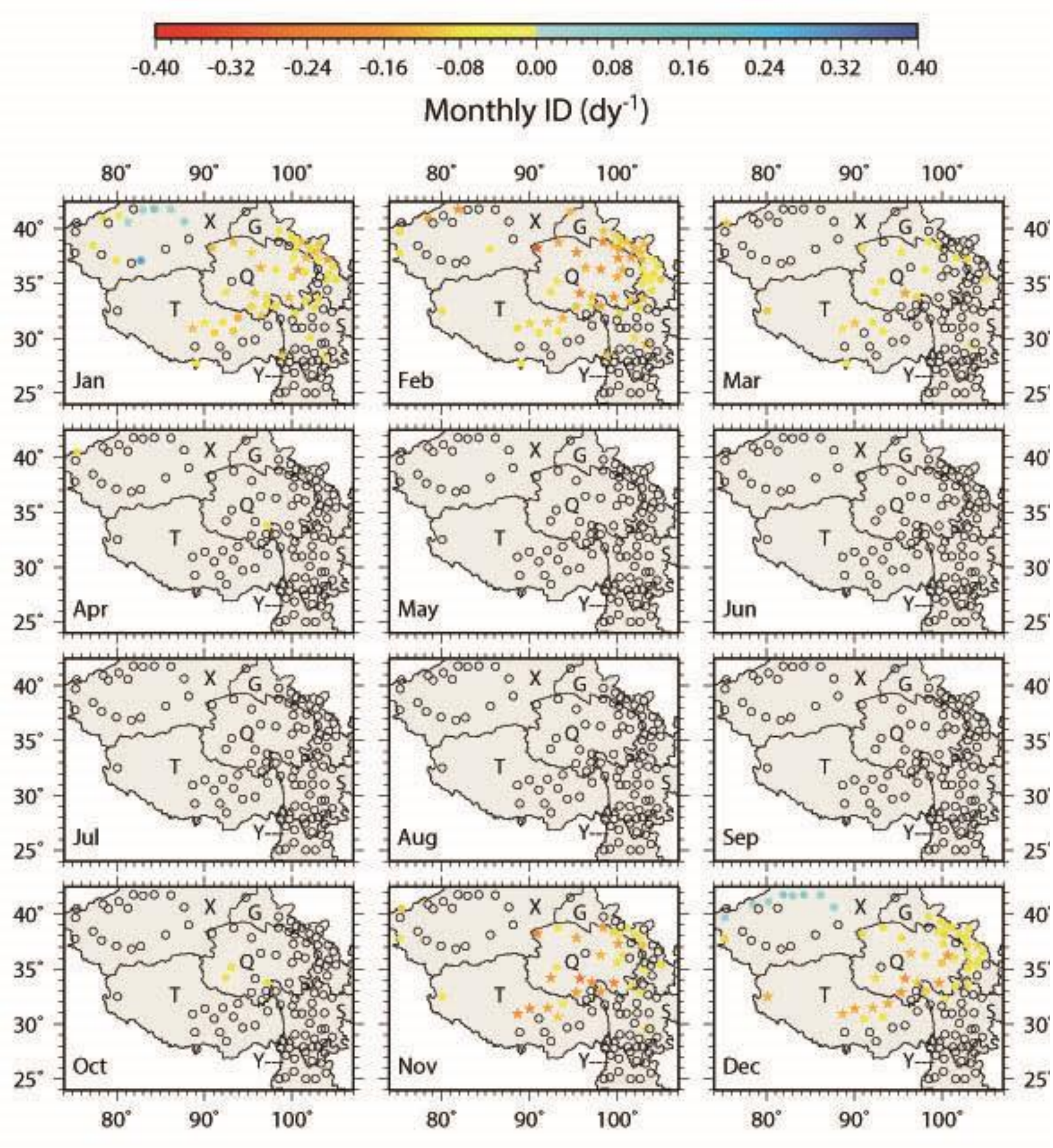

Figure S11. The spatial distributions of the trends of monthly icing days during 1963-2015. Black circles represent zero trends. Stars represent statistically significant trends ($p<0.05$).

Statistically significant negative trends of ID are noted in Q, the central G and T, and the western X from November to February, especially at Qumalai (56021, -0.2727 dy^{-1} in November and -0.2143 dy^{-1} in February) and Mangai (51886, -0.3095 dy^{-1} in February and -0.2045 dy^{-1} in November) in Q, and Bange (55279, -0.2000 dy^{-1} in November) in T.

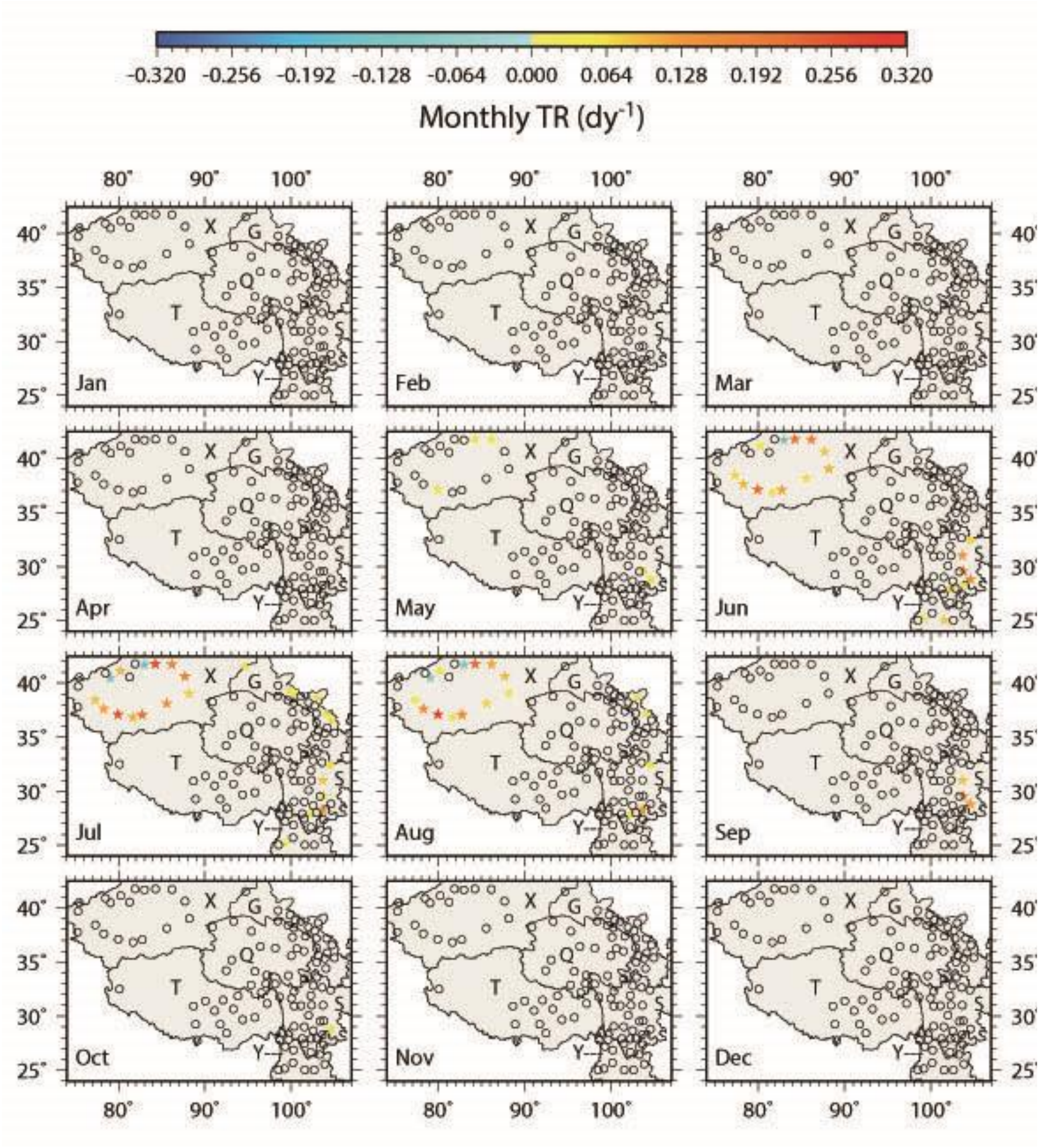

Figure S12. The spatial distributions of the trends of monthly tropical nights during 1963-2015. Black circles represent zero trends. Stars represent statistically significant trends ($p<0.05$).

Statistically significant TR trends occur primarily in X in June - August, e.g., Kuche (-0.1463 dy^{-1} in July, -0.1290 dy^{-1} in June and -0.1154 dy^{-1} in August) and Keping (51720, -0.1429 dy^{-1} in July and -0.0833 dy^{-1} in August); Hetian (51828, 0.3170

dy^{-1} in August, 0.2631 dy^{-1} in July and 0.2222 dy^{-1} in June), Luntai (51642, 0.2632 dy^{-1} in July, 0.2222 dy^{-1} in August and 0.2121 dy^{-1} in June), Minfeng (51839, 0.2069 dy^{-1} in July and 0.1667 dy^{-1} in August), Tieganlike (0.1852 dy^{-1} in July) and Luerle (51656, 0.1786 dy^{-1} in June and 0.1777 dy^{-1} in July).
